# Substituent‐Based Modulation of Self‐Assembly and Immunogenicity of Amphipathic Peptides

**DOI:** 10.1002/advs.202518567

**Published:** 2026-01-20

**Authors:** Anirban Das, Ushasi Pramanik, Elise M. Brown, Chih‐Yun Liu, Huan Gong, Jonathan Fascetti, Mark Gibson, Samuel Stealey, Silviya P. Zustiak, Cory Berkland, Piyoosh Sharma, Meredith E. Jackrel, Mark A. White, Jai S. Rudra

**Affiliations:** ^1^ Department of Biomedical Engineering Washington University in St. Louis St. Louis Missouri USA; ^2^ Department of Chemistry Washington University in St. Louis St. Louis Missouri USA; ^3^ Department of Pharmaceutical Chemistry The University of Kansas Lawrence Kansas USA; ^4^ Department of Biomedical Engineering Saint Louis University St. Louis Missouri USA; ^5^ Sealy Center for Structural Biology and Molecular Biophysics & Department of Biochemistry and Molecular Biology University of Texas Medical Branch Galveston Texas USA

**Keywords:** antibody, aromatic substitution, immune response, nanofiber, peptide, self‐assembly

## Abstract

Self‐assembled peptide‐based biomaterials provide versatile platforms for biomedical uses, featuring customizable physicochemical properties, biocompatibility, and dynamic capabilities. This self‐assembly process is primarily dictated by primary sequence features, such as hydrophobicity, length, and charge, leading to the formation of fibrils and hydrogels. Amphipathic peptides, with alternating polar and hydrophobic residues, are especially effective in forming supramolecular nanofibers stabilized by π–π interactions and hydrogen bonds. Chemical modifications on aromatic side chains are promising for controlling assembly morphology, stability, and biological activity. However, the influence of these substituents on peptide packing and immunogenicity remains relatively unexplored. Herein, we examine the effect of substituents on benzyl groups attached to short amphipathic peptides. By introducing different electron‐donating and withdrawing groups at the para‐position of benzyl rings and modifying the chain length connecting the backbone to the aromatic moiety, we observe notable effects on fibril formation, molecular packing, and immunogenicity both in vitro and in vivo. Our results show that subtle chemical modifications are practical tools for designing tailored peptide nanomaterials with promising potential in vaccine delivery, tissue engineering, and regenerative medicine.

## Graphical abstract

This study systematically investigates how positional and subtle changes, such as substituents on the phenyl ring attached to short amphipathic peptides, influence their self‐assembly, fibril morphology, and immunogenic responses.

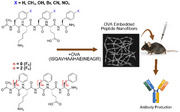

## 1 Introduction

Peptide‐based biomaterials developed through monomer‐by‐monomer self‐assembly offer significant advantages due to their unique physicochemical properties, biocompatibility, and dynamic characteristics [1]. Peptides serve as excellent building blocks due to the chemical diversity of amino acids, allowing for precise control over self‐assembly triggers such as pH, temperature, and ionic strength [2, 3, 4]. By tailoring primary sequence features, including hydrophobicity, length, chirality, and charge, various superstructures, such as micelles [5], nanofibers [6, 7, 8, 9], nanotubes [10, 11, 12], and vesicles [13], can be engineered to possess specific properties. Amphipathic designs with alternating polar/charged and hydrophobic residues readily self‐assemble into high‐aspect‐ratio cross‐β fibril nanofibers in aqueous buffers [14]. These fibers entangle at sufficiently high concentrations to form self‐supporting hydrogels with numerous biomedical applications. In particular, (FKFE)_n_ repeat sequences featuring phenylalanine (F) as the nonpolar component are well‐characterized, with abundant data relating to sequence length and variation patterns of hydrophobic and charged residues on self‐assembly [15, 16]. The minimal length, strong assembly propensity, and gelation properties of KFE8 (double repeat of FKFE) have made it the most thoroughly studied member of this peptide class [17, 18, 19].

The stability of supramolecular peptide assemblies relies on a delicate balance of non‐covalent interactions, primarily hydrogen bonding, π‐π stacking, electrostatic, van der Waals forces, steric repulsions, and hydrophobic interactions [20, 21]. Studies show that removing a single terminal residue from KFE8 significantly alters, and under certain conditions, completely prevents self‐assembly. In recent years, employing substituent groups (such as halogens, alkyls, and alcohols) to disrupt this equilibrium and promote assemblies with diverse scales and morphologies has emerged as a significant area of research in material science and engineering [4, 22]. Substituent effects are also of great interest to chemists, finding applications in supramolecular chemistry to modulate the structures of metal coordination cages, polymers, and metal‐organic frameworks (MOFs) [23, 24]. Notably, substitutions involving activating electron‐donating groups (EDGs) or deactivating electron‐withdrawing groups (EWGs) on benzyl rings have been extensively studied in organic chemistry. The subtle changes in electron cloud distribution due to substituent effects significantly impact the reactivity, stability, and biological activity of the molecule, thereby influencing its structure and interactions with other molecules. Furthermore, the position of the substituent on the ring (ortho, meta, or para) can affect the packing structure and the molecules’ orientation.

Given the essential role of hydrophobic residues in the self‐assembly of amphipathic peptides, Nilsson and colleagues leveraged the abundance of benzyl groups in (FKFE)_n_ peptides to create a valuable framework for understanding how substituent groups such as CH_3_, Cl, Br, F, OH, NO_2_, and CN influence π–π interactions in self‐assembly or co‐assembly processes [4]. He further stated the importance of local dipolar interactions between elements of neighboring aromatic rings in stabilizing π−π interactions that lead to fibril formation in EWG/EDG‐substituted peptides. Also, substitution with electron‐withdrawing groups (NO_2_, CN, F) is found to perturb the quadrupole electronics via π‐resonance and local dipole electronics inductively through the σ‐bond framework and via‐space field effects. Electron‐withdrawing groups primarily reduce the overall negative charge of the π‐system. Substitution with electron‐donating groups (NH_2_, OH, CH_3_), on the contrary, enhances the negative quadrupole of the aromatic ring in addition to the local dipole effects. This was also supported by Cozzi and Siegel's polar/π model [25], and the Hunters and Sanders model [26, 27]. However, prior studies relied on minimal systems consisting of a single F residue or diphenylalanine (FF) peptides linked to a fluorenyl methoxycarbonyl (Fmoc) protecting group [28, 29]. The reported crystal structure of Fmoc‐FF by Adams and coworkers shows that π‐stacking interactions between Fmoc groups and side chain phenyl groups, along with the hydrogen bonding interactions among carbamate and amide groups, are key drivers of self‐assembly [30]. Other studies using natural amyloid sequences (KLVFF) [31, 32] or designed peptides (FQFQFK) [33, 34] focused solely on halogenation to modulate assembly kinetics, morphology, and gel stiffness. A significant knowledge gap exists in understanding how substituents on the benzyl group influence the assembly and molecular packing of F‐rich peptides that lack Fmoc protecting groups. Moreover, no studies have reported the in vivo effects of substituent modifications on the (FKFE)_n_ class of peptides, making it crucial to address this gap, as insights from these studies could lead to the development of higher‐order and multi‐component hydrogels for biomedical applications.

**SCHEME 1:**
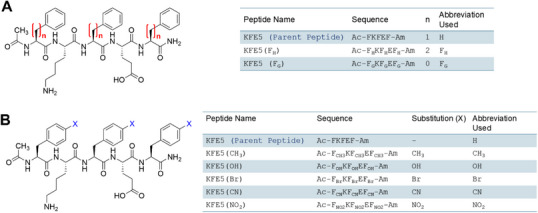
Sequences of KFE5 (A) positional variants, and (B) chemical variants used in this study.

In this study, we utilized KFE5 (Ac‐FKFEF‐Am) (Scheme 1, Figures S1–S8), a truncated variant of KFE8 (Ac‐FKFEFKFE‐Am), to investigate the effects of substituents on the self‐assembly and immunogenicity of supramolecular peptide nanofibers. KFE5 was identified as the minimal sequence necessary for self‐assembly, as no fibril formation was observed with KFE4 (FKFE). We also varied the distance of the benzyl group from the backbone by replacing F with either the more rigid phenylglycine (F_G_) or the more flexible homophenylalanine (F_H_). This resulted in the benzyl group being placed at zero carbon distance (n = 0, F_G_), one carbon distance (n = 1, F), or two carbon distance (n = 2, F_H_) from the peptide backbone (Scheme 1A). We introduced EWGs (Br, CN, NO_2_) or EDGs (CH_3_, OH) at the para position (Scheme 1B) to assess their impact on self‐assembly. Thorough biophysical and biochemical characterization utilizing various spectroscopic techniques revealed a fundamental β‐sheet‐rich fibrillar nature with diverse molecular packing modes, morphologies, and bulk rheological properties. Cross‐seeding assays with FRET biosensor cells demonstrated that the constructs are non‐toxic and non‐amyloidogenic. Additionally, treating primary bone marrow‐derived dendritic cells (DCs) with modified nanofibers resulted in differing innate immune activity. RNA‐seq analyses in primary DCs indicated upregulated stress response pathways, and all constructs were compatible with modification using the model antigenic peptide OVA_323‐339_ (ISQAVHAAHAEINEAGR). In mice, substituent variations led to the development of differential antibody and cellular immune responses. Our findings suggest that substituents offer a wider range of design blocks for constructing synthetic peptide assemblies, serving as a straightforward yet potent tool for creating custom biomaterials in engineering and medicine.

## 2 Results

### 2.1 Self‐Assembly of KFE5

**FIGURE 1:**
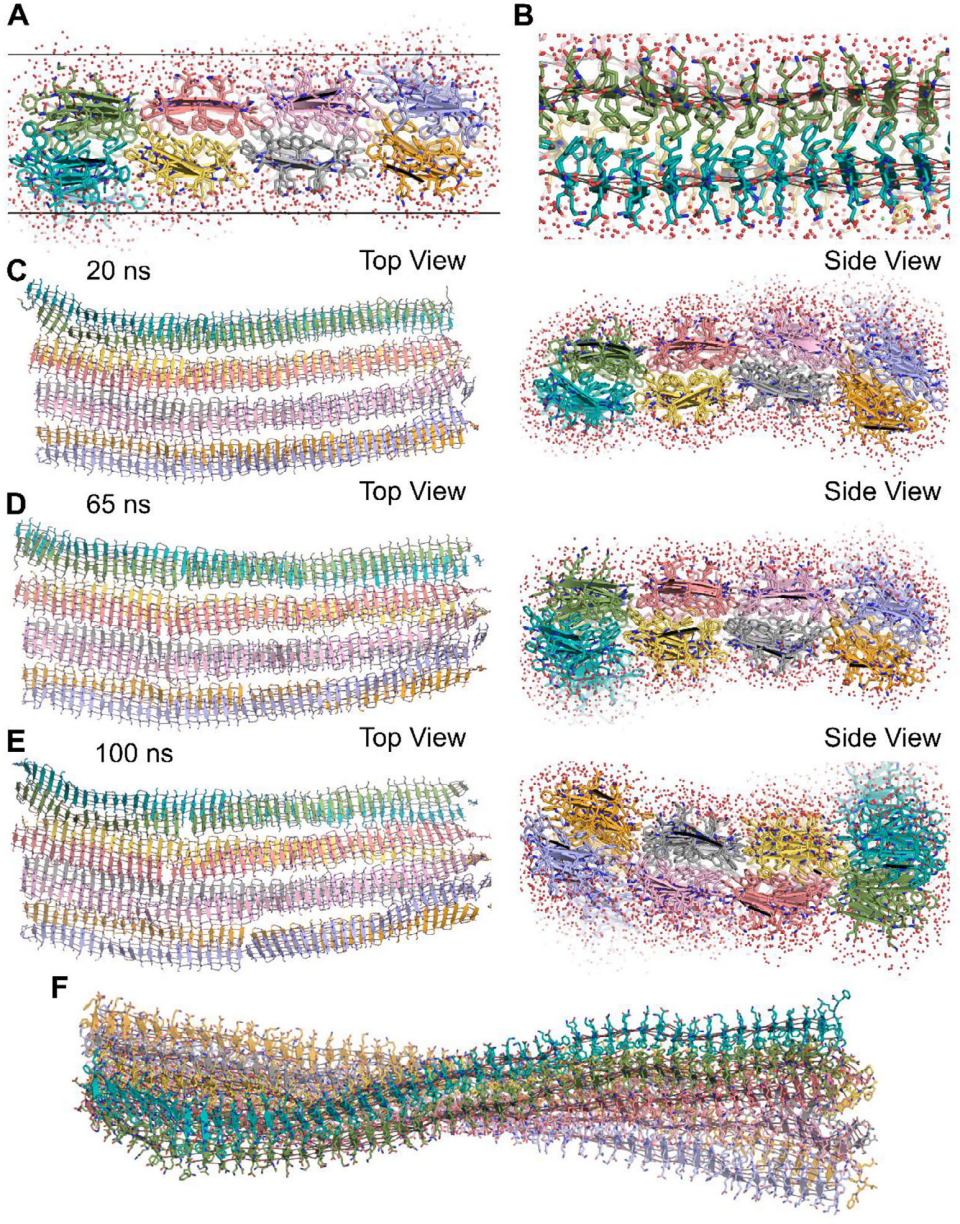
A model of the KFE5 assembly. (A) Close‐up view of the KFE5 peptide assembly. The solvent layer around the peptide is shown. The two horizontal lines demark the circa 32 Å sheet thickness determined via SAXS. (B) Edge‐on view of the assembly showing the phenylalanine hydrophobic core. The β‐sheet‐forming hydrogen bonds are shown as lines. (C,D,E) KFE5 4‐fibre Tape model (4× (48 × 2)) Tape Model at respective time points using free MD run, as viewed down the sheet/Tape normal, along with their view along the fiber axis. (F) The 384‐peptide cartoon model of the two fibers interacting through their mixed hydrophobic and charged edges (Side‐chains not shown for clarity).

The assembly of the pentapeptide FKFEF in water was characterized using negative‐stain TEM. Data shows that compared to KFE8 (FKFEFKFE), which assembles into thin nanofibrils (8–10 nm wide), the truncated variant KFE5 (FKFEF) exhibits markedly different assembly behavior, forming broader tape‐like structures 50–100 nm wide (Figure S9). This significant increase in fibril width is attributed to a shift in the register of amino acid alignment during the assembly process. Notably, in KFE8, the terminal phenylalanine is out of register, which influences the stacking interactions and stabilizes the formation of narrow nanofibers [35]. In contrast, the shorter KFE5 lacks this out‐of‐register terminal phenylalanine, leading to altered intermolecular interactions and a different registry of amino acids within the fibril core. This shift affects the packing density, favoring the formation of wider tapes rather than the narrower nanofibers observed in KFE8. To gain insights into KFE5 assembly, we employed molecular dynamics (MD) simulation of KFE5 based on an Alphafold3 prediction and the model previously developed for KFE8 (Figure S10) [36]. The Alphafold3 predictions for 30 copies of KFE5 included one anti‐parallel 2‐start fiber, similar to the KFE8 model previously reported by us [14, 36]. In this model, significant interactions contributing to the self‐assembly of the pentapeptide are the π‐π stacking interactions between F‐F in a hydrophobic core, sandwiched between the β‐sheet‐forming peptide backbone (Figure 1A,B). As described in the methods section, we extended this initial 30‐peptide proto‐fiber to a longer 192‐peptide dual‐fiber model for analysis using molecular dynamics (Figure 1C–H).

The KFE5 fiber has the basic configuration of the KFE8 and KFE12 peptide assemblies [37], featuring a hydrophobic core of phenylalanine sandwiched between the β‐sheet‐forming peptide backbone. One significant difference with KFE5 is the alignment of the phenylalanine residues along the edge of the fiber. This produces a hydrophobic strip along the edge of each fiber sandwich, creating a source for inter‐fiber interactions. The alignment of the upper and lower sheets varies throughout the simulation, leaving an overlap at each edge that could allow other fibers (Figure 1I) to join the assembly, which can now grow in both directions, along the fiber axis (z‐plane) and in the plane of the β‐sheet (x‐plane). The edge interactions are almost exclusively hydrophobic and exclude solvent atoms from the fiber interface. These findings underscore the importance of peptide sequence length and amino acid register in modulating the morphology and dimensions of self‐assembled peptide nanostructures.

### 2.2 Substituent Effects on the Self‐Assembly of KFE5

**FIGURE 2:**
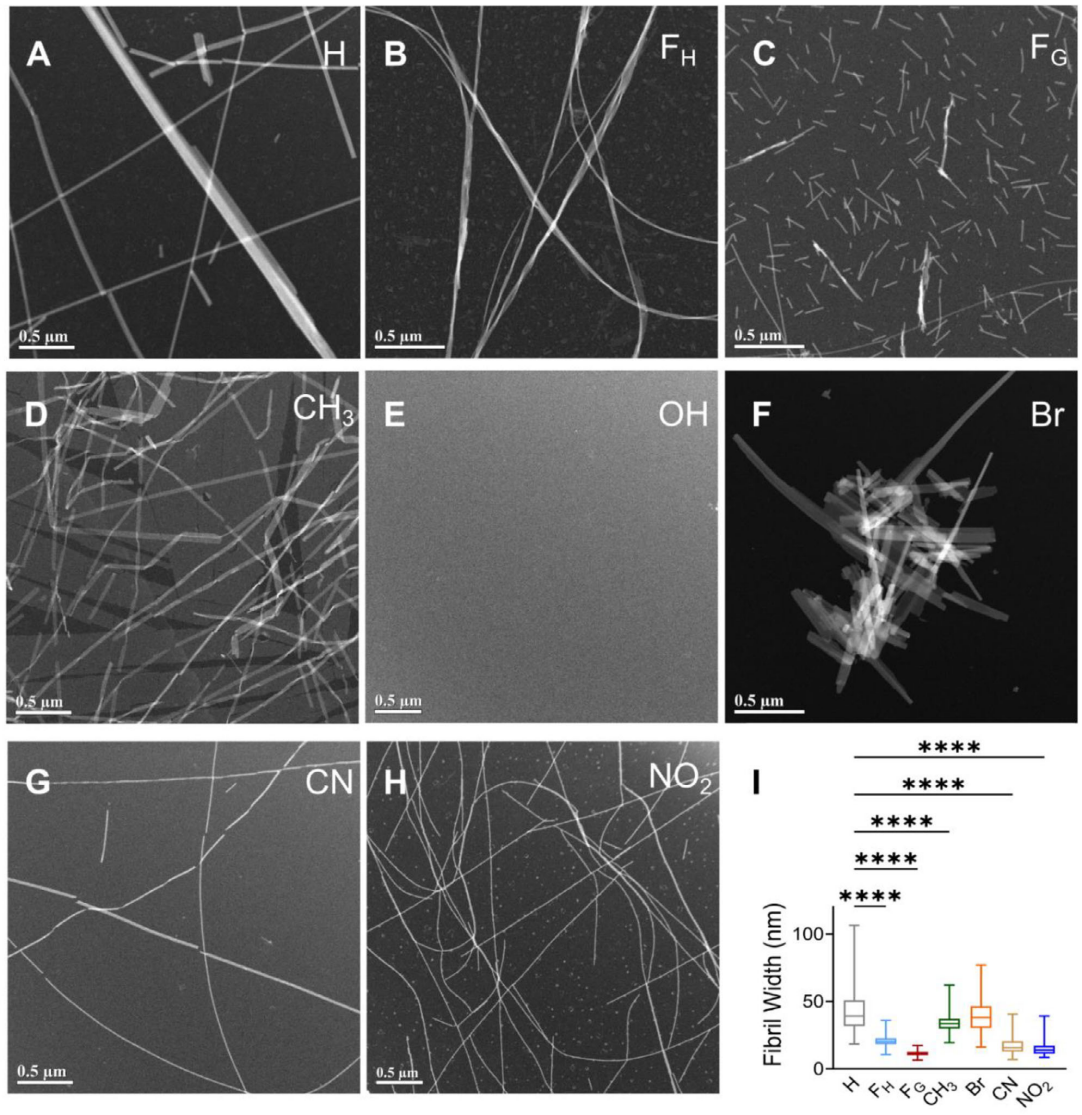
Representative TEM images of self‐assembled pentapeptides having different substitutions. (A) H, (B) F_H_, (C) F_G_, (D) CH_3_, (E) OH, (F) Br, (G) CN, (H) NO_2_ with difference in (I) fibril width as calculated from TEM images (n = 200 measurements from 30 fibrils). ****p < 0.0001 as determined by a one‐way ANOVA.

In water and under identical preparation conditions, all peptides, except the hydroxyl (OH)‐substituted variant, assembled into 3D nanostructures (FigureS 2A–H, S11). Morphological differences among the fibrils were evident as the electronic properties of the substituents attached to the benzyl group modulate the strength of the π‐π interactions, molecular packing, and the overall fibrillar architecture. EWGs decrease the negative charge density on the π‐cloud, while EDGs exert the opposite effect via local dipole interactions, altering the aromatic quadrupole. In stark contrast with the unsubstituted parent peptide (X = H), which formed 50–100 nm wide tapes, the presence of EWGs, CN, and NO_2_ resulted in fine and significantly thinner fibrils (∼15–17 nm wide). Interestingly, bromination resulted in dense, wide tapes with similar widths (∼40 nm wide) compared to the parent peptide KFE5.

Substitution with the EDGs, OH, and CH_3_ led to complete ablation or formation of dense twisted fibrils, respectively. These morphological diversities are likely due to the relative strengths of the EDGs, with the strong resonance active OH group inhibiting assembly and the weaker inductive electron donor CH_3_ promoting assembly, especially at the para position. The width of the methylated fibrils was comparable to the parent peptide (∼42 nm), and they were the only fibrils that exhibited a twisted morphology. Further, lengthening (F_H_) or shortening (F_G_) the benzyl group position from the peptide backbone also resulted in notable changes in fibril morphology. The F_G_ variant formed shorter fibrils, averaging 117 ± 56 nm in length, whereas the F_H_ variant produced bundled thick fibrils with similar lengths compared to the KFE5. Interestingly, the fibril width was decreased for both F_H_ and F_G_ peptides, with the F_G_ peptides displaying the lowest width (15 nm). These differences probably arise from variations in side‐chain volume and orientation within the hydrophobic bilayer, which influence peptide packing and assembly. The impact of these structural variations on fibril width is summarized in Figure 2I.

### 2.3 Determination of Secondary Structure

**FIGURE 3:**
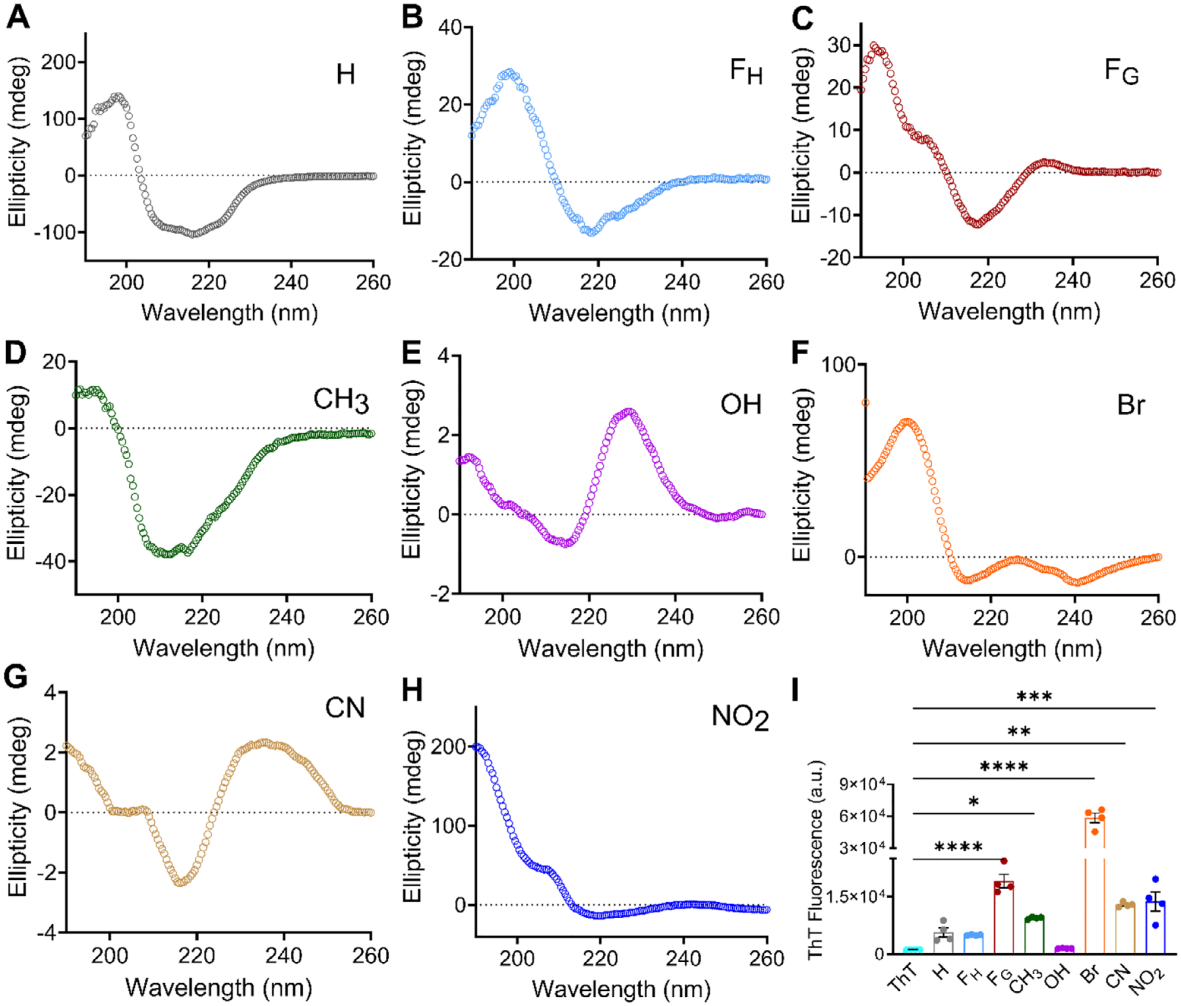
Secondary structure determination of synthesized peptides used in the study. (A–H) CD spectra of self‐assembled pentapeptides showing differences in secondary structures. (I) Variations in ThT fluorescence intensities in the presence of different fibrils formed by pentapeptides, as marked in the figure (n = 4). *p < 0.05, **p < 0.01, ***p < 0.001, ****p < 0.0001 as determined by a one‐way ANOVA.

Circular dichroism (CD) spectroscopy data indicate that KFE5 fibrils adopt a classical β‐sheet signature, characterized by a negative peak centered around 220 nm (n–π* transition) and a positive peak near 198 nm (π–π* transition) (Figure 3A). Variations in the β‐alkyl chain length modulated the spectral broadness; notably, the broad minima observed between 210–220 nm in the parent peptide shifted to sharper minima centered at ∼218 nm for both F_G_ and F_H_ (Figure 3B,C). The CH_3_‐substituted analog retained the canonical β‐sheet CD signature of the KFE5 peptide (Figure 3D). A strong positive CD signal was detected at ∼235 nm for the OH‐substituted peptide (tyrosine) despite the lack of fibril formation observed by TEM (Figure 3E). This is a hallmark of **tyrosine aromatic stacking interactions** in peptide fibrils or aggregates, serving as a spectroscopic marker of such interactions [38]. Substitutions with EWGs induced a shift in the CD spectra, with the brominated peptide displaying a negative ellipticity peak ∼240 nm (Figure 3F), attributable to electronic transitions associated with the amide groups and aromatic interactions within β‐sheets. The CN and NO_2_ peptides, on the other hand, exhibited a positive Cotton effect [39] at 240 nm, associated with π–π stacking and aromatic stacking interactions (Figure 3G, H). Using BeSTSel analysis, we deconvoluted the CD spectra for different peptides and found the presence of anti‐parallel β‐sheet as the primary secondary structure in all the peptide fibrils, with significant contribution also arising from parallel β‐sheet orientations. Specifically, for the F_H_ peptide, the formation of the parallel β‐sheet was found to be more than the anti‐parallel one (Table S1).

To confirm the formation of β‐sheet‐rich fibrils, Thioflavin T (ThT) fluorescence assays were performed. ThT, a well‐established amyloid fibril marker [40, 41, 42], exhibits increased fluorescence upon binding to β‐sheet‐rich structures due to restricted bond rotation. 50 µM peptide was used to compare the ThT (10 µM) fluorescence intensity amongst the different peptide variants (Figure S12), since the ThT fluorescence was found to be saturated beyond this concentration of peptide fibrils. The brominated peptide produced the highest ThT fluorescence (Figure 3I), commensurate with the dense thick fibrils observed by TEM and the β‐sheet structure revealed by CD spectroscopy. The presence of EWGs CN and NO_2_ also led to an increased ThT signal, which was less than that of the strong halogen Br but higher than that of the parent peptide. Consistent with TEM data, the signal from the OH‐substituted variant was the lowest and comparable to ThT alone, suggesting a lack of fibrils, whereas CH_3_ substitution promoted fibril formation and increased fluorescence. Interestingly, despite forming fibrils of lower length and width, fluorescence was enhanced for the F_G_ peptide compared to the parent peptide or the F_H_ peptide.

Complementing our CD data, FT‐IR analysis of the substituted peptides revealed predominantly β‐sheet characteristics, with major amide‐I bands centered at 1623–1627 cm^−1^ (Figure S13). The additional peak, centered around 1679–1687 cm^−1^, represents an anti‐parallel β‐sheet orientation in almost all the peptides. Fibrils formed from F_G_ peptide, however, do show a significant amount of disordered nature along with β‐sheet characteristics, with a sharp peak at 1647 cm^−1^. Similarly, the anti‐parallel β‐sheet orientation, with peaks centered around 1682 cm^−1^, is almost absent from the fibrillar pattern observed in the F_H_‐substituted peptide, suggesting the formation of a parallel β‐sheet in this case and further corroborating our results from CD spectroscopy and the deconvoluted data. Thus, the FT‐IR analysis further confirms the β‐sheet‐rich secondary structure for all the fibrils formed by different pentapeptides.

### 2.4 Substituent Effects on Viscoelastic Properties of Peptide Hydrogels

**FIGURE 4:**
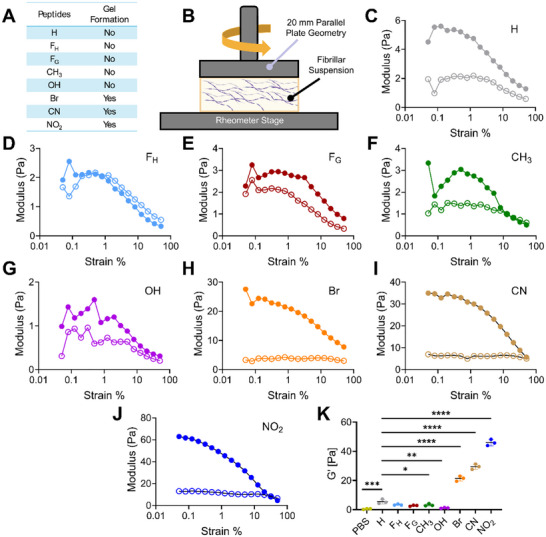
Viscoelastic properties of peptide hydrogels. (A) Probability of gel formation by peptides with substitutions Br, CN, and NO_2_ at 5 mM peptide concentration, as compared to the parent peptide and other KFE5 variants. (B) Schematic of peptide nano‐fiber solution testing using a rotational rheometer. (C–J) Representative plots showing the evolution of G′ (dark, solid circles) and G″ (light, open circles) as a function of strain amplitude. (K) G′ of peptide solutions at a strain of 1% and an angular frequency of 10 rad/s. *p < 0.05, **p < 0.01, ***p < 0.001, ***p < 0.0001 as determined by a one‐way ANOVA (n = 3).

The gelation propensity and viscoelastic behavior of peptide hydrogels with various substituents were characterized (Figure 4A,B). Data indicated that despite robust assembly and a high β‐sheet content, a 5 mM solution of the parent KFE5 peptide failed to form a solid gel (Figure 4A). However, all peptide variants bearing EWGs (Br, CN, NO_2_) formed self‐supporting gels at the same concentration. In contrast, peptides substituted with EDGs (OH and CH_3_) also failed to undergo gelation at a peptide concentration of 5 mM (Figures 4A, S14A) [43]. TEM images confirmed the network‐like fibrillar structures for all the EWG‐substituted peptides (Figure S14B). Gel‐forming derivatives exhibited viscoelastic properties, with the storage modulus (G′) significantly exceeding the loss modulus (G″), whereas non‐gelling substitutions showed less significant differences between G′ and G″ (Figure 4C–J). Notably, NO_2_ substitution derivatives displayed the highest G′ values (∼eightfold higher than parent peptide), whereas OH substitution resulted in a G′ approximately 2.5‐fold lower than the parent peptide, as expected. Substitution with CH_3_ yielded intermediate G′ values.

Complementary viscosity measurements corroborated these rheological trends, showing that the parent peptide possessed the highest viscosity among the non‐gelling derivatives, whereas OH substitution resulted in the lowest viscosities (Figure S14C). EWG derivatives (Br, CN, NO_2_) demonstrated viscosities at least threefold higher than the parent peptide. These findings are supported by TEM imaging, which revealed morphological differences consistent with the rheological data. The side chain length also influenced mechanical properties; peptides with an extended phenyl group (F_H_), formed thicker and longer fibrils, correlating with increased G′ and viscosity. These F_H_‐hydrogels exhibited shear‐thinning behavior, similar to that of F_G_ substitutions, indicating a potential for injectable applications. Overall, substituent‐induced variations in fibril morphology directly impacted the viscoelastic properties of the peptide hydrogels, revealing the capacity to tune mechanical characteristics through side chain chemistry (Figure 4K).

### 2.5 Small‐Angle X‐ray Scattering and Wide‐Angle X‐ray scattering (WAXS) Analysis of the 5‐Mer Peptide Assemblies

**FIGURE 5:**
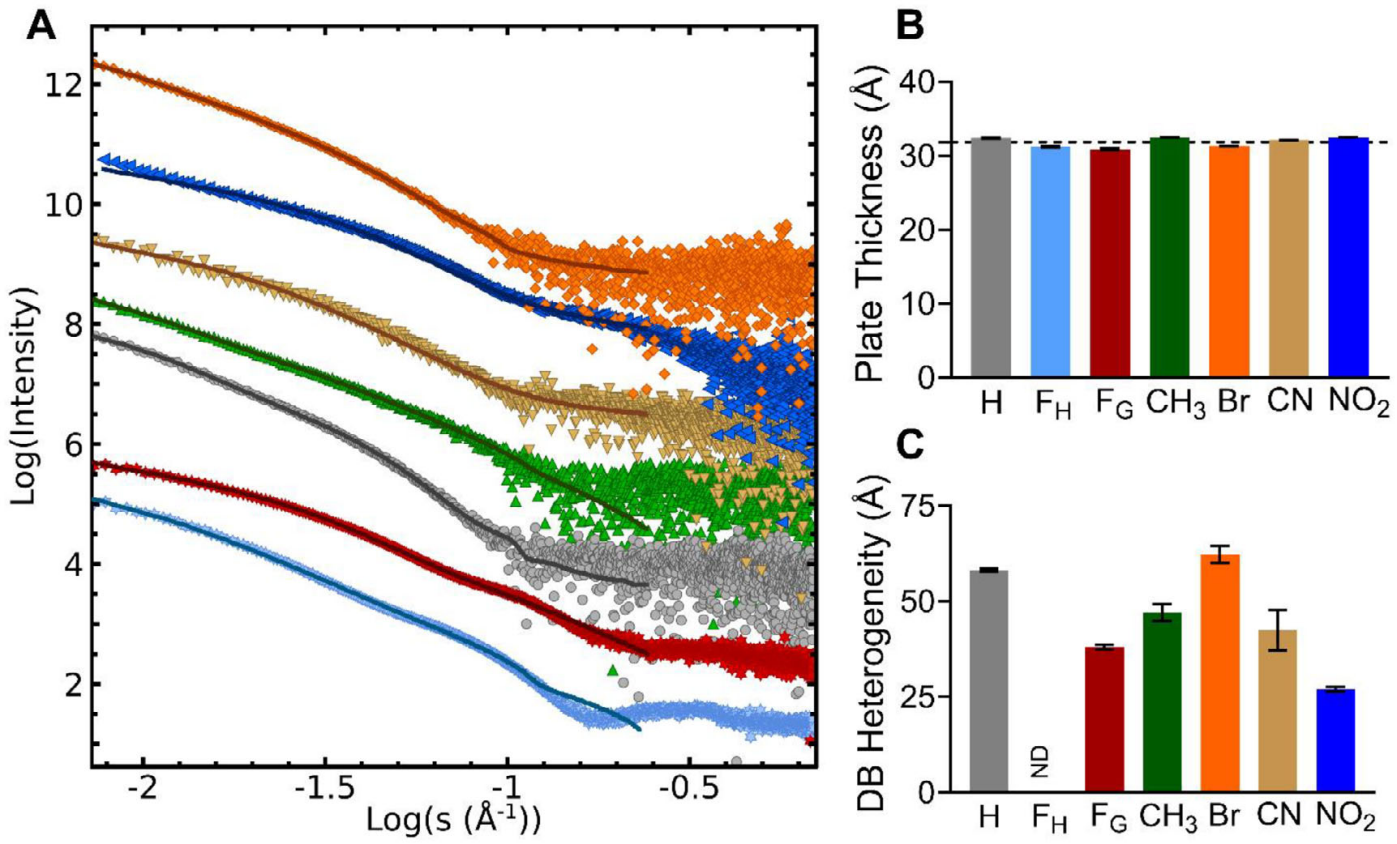
SAXS analysis of 5‐mer peptides. (A) The Log‐Log 4 mg/mL SAXS curves for (◆) Br, (▼) CN, (◀) NO_2_, (▲) CH_3_, (⬤) H, (✶) F_G,_ and (✶) F_H_. SAXS curves are offset for clarity. (B) The GNOM Debye P(r) plate thickness fitting. The Debye P(r) was fit to a 6‐term Fourier series with an exponential amplitude decay. The average Plate thickness, τ∼31.9(1) Å, is shown as a black dotted line. (C) The ζ for the KFE5 peptides. The value of ζ for the F_H_‐substituted peptide was undetermined.

All the KFE5 peptide SAXS curves fit simple shape models of long polydisperse rods plus smaller globular components (Figure 5A). The characteristics of these rods, extracted from fitting to a Guinier‐plate model, are almost identical with an average rod or tape thickness estimated at 31.9(1) Å (Figure 5B). This is similar to our previously measured values for KFE8 and KFE12 [14, 37]. This thickness, which includes contributions from the solvent layer, matches the β‐sheet sandwich model. This appears to be a common feature of the KFE peptides that is not disrupted by the modification of the phenylalanine side‐chain, which forms an oily interface between the upper and lower β‐sheets (Figure 1A). The width of these tapes, as measured by TEM (Figure 2I), is at the limit of resolution of our SAXS data, about 60 nm (600 Å). Therefore, we cannot apply the standard Guinier radius of gyration, Guinier‐Rod radial cross‐section approximations, or Holtzer‐plot analyses to the SAXS data, but the Guinier‐plate analysis, which models an infinite sheet, is expected to be unaffected by this. The Debye‐Bueche model of gel n‐homogeneity is also likely to be unaffected. The normalized residuals of Debye‐Bueche inhomogeneity‐correlation length (ζ) plots (SAXS plots fitted to DB equation) have reasonable but systematic variations with minor errors in ζ, except for F_H_, which was undetermined, indicating that the approximation is valid over the selected fitting range (0.015–0.10 Å^−1^). The gel inhomogeneity‐correlation length (ζ) of the KFE5 peptides was similar (Figure 5C) in the range of 27 Å for NO_2_, 38–47 Å for CN, F_G_, and CH_3_, and about 58 Å for H and Br. The ζ of F_H_ was not well‐determined, since the SAXS curve was not well‐fit by the Debye‐Bueche model. These compare with ζ values of 25 Å for the KFE8 peptide and 17 Å for KFE12, representing a trend toward larger ζ values for the shorter peptides.

X‐ray diffraction scattering relies on a regular repeat of features in a molecular assembly and is an ideal tool for fibril‐forming peptides such as KFE5. The β‐sheet peak at d∼4.7 Å was the strongest peak in the X‐ray diffraction (XRD) curves for all the KFE5 peptides (Figure S15). This phenomenon has also been observed in studies of other KFE peptides, specifically those of lengths 8 and 12, and is maintained in the KFE5 peptide fibers. The KFE5 peptide differs in the positioning of phenylalanine residues at both ends of the peptide, which modeling suggests aids in the formation of broad tapes formed by multiple fibers. Unique to KFE5, the second and third strongest XRD peaks are the d∼20 Å peak and the d∼24 Å peak from longer‐range ordering of the fibers (Figure S15, Table S2). However, in the modified phenylalanine peptides, these peaks are significantly weaker, dropping from ∼40% of the β‐sheet peak intensity in KFE5 to 11% for CN, and 8% or lower for the other modified phenylalanine peptides. The shorter distance of 20 Å corresponds to the edge‐to‐edge fiber repeat distance (perpendicular to the fiber axis) in the KFE5 tape packing model. The weaker 24 Å peak matches the diagonal inter‐fiber distance between the upper and lower β‐sheets. The modified phenylalanine peptides have weaker edge fiber repeat peaks, indicating that they are less ordered along the inter‐fiber tape‐forming direction.

### 2.6 Effects of Chemical Substituents on Innate Immunity

Prior studies using KFE8 and other self‐assembling peptide nanofibers have shown that they activate innate immune cells, such as dendritic cells (DCs) and macrophages, via the release of damage‐associated molecular patterns (DAMPs) [44, 45]. Studies have reported the upregulation of maturation markers, such as MHC II, CD80, and CD86, along with the secretion of chemokines (MCP‐1α/CCL2, KC/CXCL1) and cytokines (GM‐CSF, IL‐5, IL‐6, IL‐1β), in response to treatment with β‐sheet‐rich peptide nanofibers [46]. To address the effects of substituents on activation of innate immunity, we employed BMDC cultures and measured cytokine and chemokine release in response to treatment with the nanofiber variants. LPS (lipopolysaccharide), a component of bacterial cell walls and a potent activator of TLR4, was used as a positive control. Analysis of the supernatant from treated BMDCs revealed that modifications to KFE5 have a significant influence on cytokine production and immunogenicity. Physical modification with F_G_ resulted in minimal cytokine release, characterized by extremely low levels of pro‐inflammatory TNF‐α, tolerogenic IL‐10, and maturation‐associated IL‐13, along with negligible production of chemokines, including MCP‐1, MIP‐1α, MIP‐1β, and IP‐10. In contrast, F_H_ substitution led to the robust secretion of inflammatory cytokines, including IL‐6, MCP‐1, and TNF‐α, along with moderate levels of IL‐10 and MIP‐1α (Figure S16). Br substitution led to the secretion of IL‐10 and MCP‐1, IL‐6, MIP‐1α, and TNF‐α compared to the parent peptide. Conversely, NO_2_ substitution did not induce IL‐6 production, maintained MIP‐1α levels, and specifically induced MIP‐1β secretion. Of all the EWGs, the lowest activation profile was observed with CN substitution, with only moderate increases in MIP‐1α and IL‐13, similar to the unsubstituted peptide KFE5. Treatment with methylated fibrils resulted in the production of MIP‐1α, MIP‐1β, and TNF‐α. In contrast, OH substitution resulted only in the release of MIP‐1α and MIP‐1β without TNF‐α secretion, yielding a less immunogenic profile compared to CH_3_ substitution (Table S3). These findings demonstrate that the presence of substituent groups on the benzyl ring can alter the innate immune activation profiles and could have potential implications for vaccine development.

### 2.7 Substituent Effects on Amyloidogenesis and Cross‐Seeding

**FIGURE 6:**
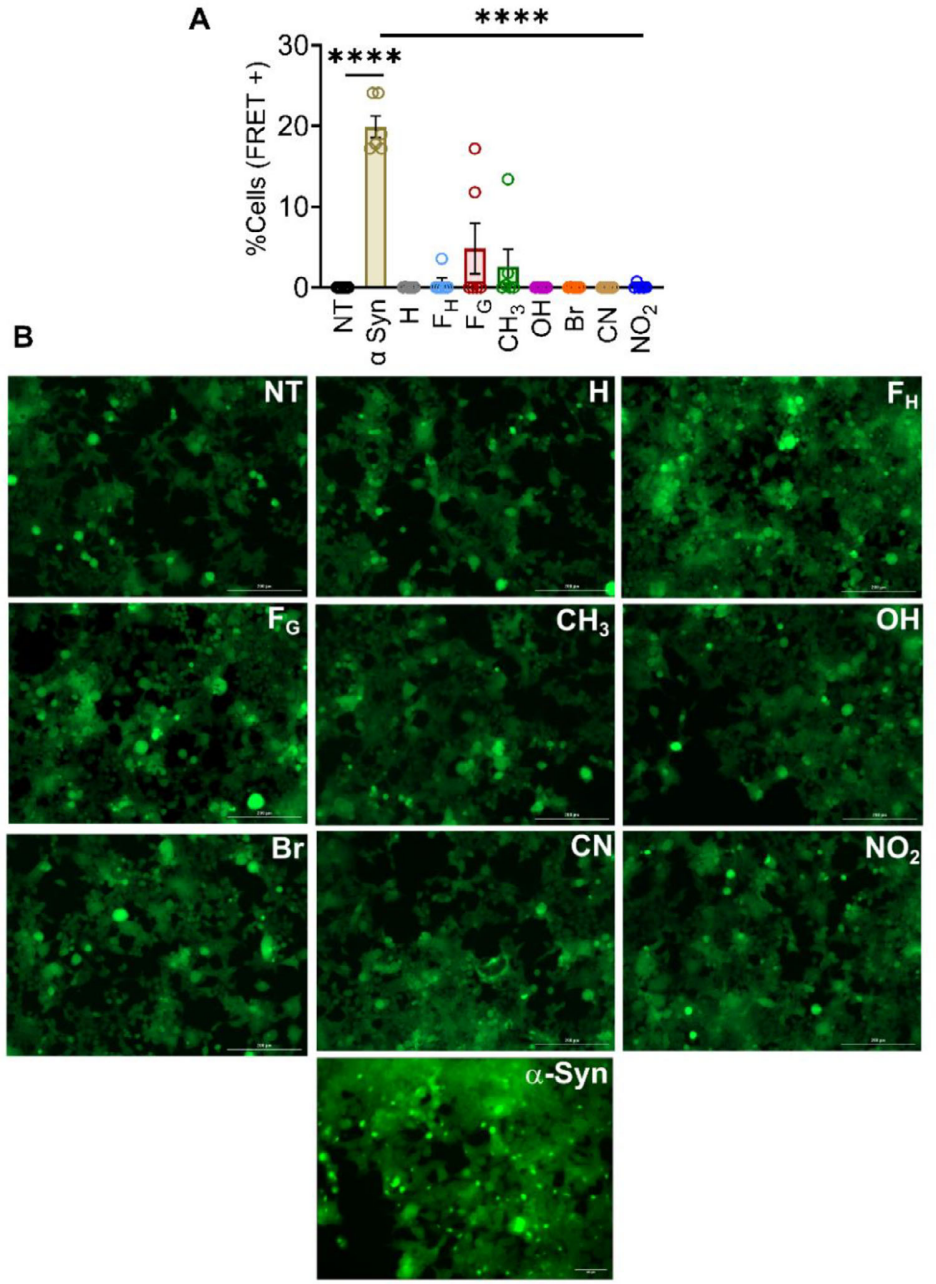
KFE5 nanofibers do not cross‐seed α‐synuclein after being internalized by HEK biosensor cells. (A) % FRET positive cells as determined by the assay at 0.5 µM nanofiber concentration compared to α‐synuclein PFFs (50 nM). *p < 0.05, **p < 0.01, ***p < 0.001, ****p < 0.0001 as determined by a one‐way ANOVA (n = 6). (B) Representative microscopy images of 50 nM α‐Syn and 0.5 µM 5‐mer PNFs added to biosensor cells. The green puncta represent FRET due to aggregation caused by α‐syn PFFs to monomeric α‐syn. Scale bar is 200 µm.

Amyloid fibrils can spontaneously enter cells and seed the aggregation of various monomeric proteins, ultimately leading to cell death [47]. Due to the structural similarity between synthetic peptide nanofibers and fibrils formed from pathological amyloids, we investigated whether substituents affect amyloidogenic propensity (Figure 6). We employed HEK293T α‐synuclein biosensor cells, in which one copy of α‐synuclein is fused to cyan fluorescent protein (CFP), while another copy is fused to yellow fluorescent protein (YFP). The CFP and YFP fused proteins, upon aggregation, can undergo fluorescence resonance energy transfer (FRET) due to their proximity. Flow cytometry quantifies this by measuring the FRET signal (Figure S17A). We found that 50 nM concentration of α‐synuclein pre‐formed fibrils (PFFs) were able to cross‐seed their monomeric form, as evident by the high percentage of cells with FRET positive signals (∼30%) (Figure S17B,C). In contrast, treatment with the peptide nanofibers did not lead to any detectable FRET signal (Figure S17B,D). These findings were corroborated using fluorescence microscopy, where abundant foci were observed upon incubating with α‐syn PFFs, but not peptide nanofibers (Figure S17D). Additionally, a tenfold excess of the peptide concentration also did not enhance FRET signals, thereby further demonstrating the non‐cross‐seeding behavior of the peptide nanofibers (Figure 6). These data suggest that nanofibers formed via designed synthetic peptides and pathological amyloids are functionally distinct despite structural similarity.

### 2.8 Immunogenicity of Antigenic KFE5 Variants

To assess the effects of substituents on the immunogenicity of the fibrils, we synthesized KFE5 variants in tandem with OVA, a peptide from chicken egg ovalbumin (aa 323–339 ISQAVHAAHAEINEAGR), which is an I‐A^b^‐restricted class II peptide (Table S4, Figures S18–S25). All OVA‐bearing variants self‐assembled into fibrils with diverse morphologies, which varied according to substitutions. The relative scarcity of fibril ends in TEM images suggested lengths on the order of microns, except for CN and F_G_ substitutions, which formed shorter fibrils (Figure S26A). The formation of β‐sheet‐rich fibrils was further confirmed by CD spectroscopy (Figure S26B).

**FIGURE 7:**
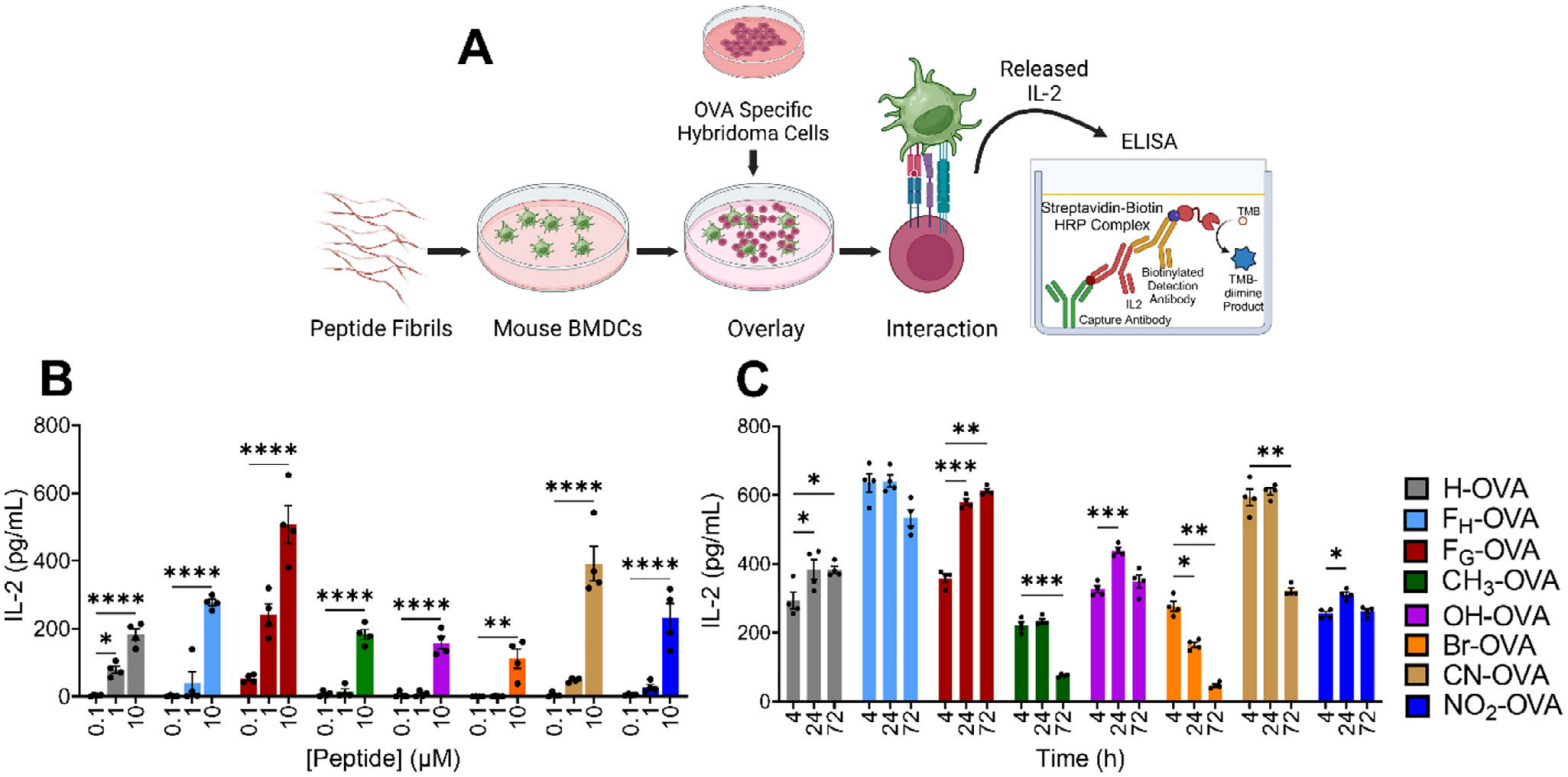
Antigenic property of KFE5 nanofibers. (A) Schematic representation depicting the ELISA assay for IL‐2 generation upon interaction of mouse BMDCs with DOBW hybridoma in the presence of 5‐mer peptide nanofibers conjugated to OVA peptide. (B) Concentration‐dependent IL‐2 production by OVA‐conjugated different peptide nanofibers upon treatment in mouse BMDCs and overlayed with DOBW hybridoma for 16 h. (C) Time‐dependent IL‐2 production by OVA‐conjugated different peptide nanofibers upon treatment in mouse BMDCs. *p < 0.05, **p < 0.01, ***p < 0.001, ****p < 0.0001 (n = 4), as determined by a one‐way ANOVA.

Antigen presentation was quantified by using DOBW hybridoma cells, which produce IL‐2 upon recognizing the OVA epitope in the context of MHC class II molecules on the surface of BMDCs (Figure 7A). The rationale underlying the method is to quantify IL‐2 production, which is directly correlated to nanofiber internalization and processing by BMDCs. An advantage of this method over flow cytometry is that it eliminates false‐positive readings caused by fibril adhesion to the cell surface. Data indicated a concentration‐dependent increase in IL‐2 production in cultures treated with OVA‐KFE5 variants (0.1–10 µM; 24 h) (Figure 7B). Differences in fibril morphology affected internalization, with shorter fibrils leading to greater IL‐2 production compared to longer fibrils. Specifically, OVA‐conjugated F_H_, F_G_, and CN variants with shorter fibril lengths induced higher IL‐2 levels, which correlated with their shorter fibril lengths from our morphological analysis (Figure S27). The only notable exception was KFE5 (Br), which showed reduced antigen presentation, even with shorter fibril lengths. The other peptides showed moderate IL‐2 production, which also increased with increasing peptide concentration (Figure 7B). We further evaluated persistence by treating BMDCs with 10 µM of OVA‐KFE5 variants, thoroughly washing, and overlaying with DOBWs at different time points to remove extracellular fibrils (Figure 7C). Data indicated an increase in IL‐2 production over time for the parent peptide and F_G_ and F_H_ variants, indicating ongoing fibril processing and presentation in the context of MHC class II molecules. Except for fibrils formed by the parent peptide and F_G_ variant, IL‐2 production decreased with time for all substitutions (Figure 7C). It is worth noting that these data also reflect the differences in internalization mechanisms and efficiency. To probe the mechanisms of cellular uptake, we employed pharmacological inhibitors targeting distinct endocytic routes (Figure S28). Before the addition of OVA‐KFE5 variants, DCs were pretreated for 60 min with inhibitors of clathrin‐mediated endocytosis (chlorpromazine, CPZ), dynamin‐dependent endocytosis (dynasore, Dyn), macropinocytosis (rottlerin, Rot), PAK1‐regulated uptake (IPA‐3), and actin polymerization (cytochalasin A, CytoA) [38]. The parent peptide KFE5, along with KFE5 (OH) and KFE5 (CN), predominantly relied on dynamin‐dependent endocytosis for cellular uptake. In contrast, OVA‐bearing KFE5 (F_H_) and KFE5 (F_G_) were primarily internalized via clathrin‐mediated endocytosis. The Br, CH_3_, and NO_2_ variants were highly sensitive to cyto A treatment, suggesting a complex internalization pathway. Collectively, these results establish a correlation between differential antigen presentation efficiency and the underlying endocytic mechanisms that govern peptide nanofiber internalization.

### 2.9 Antibody Responses to Chemically Modified Fibrils

**FIGURE 8:**
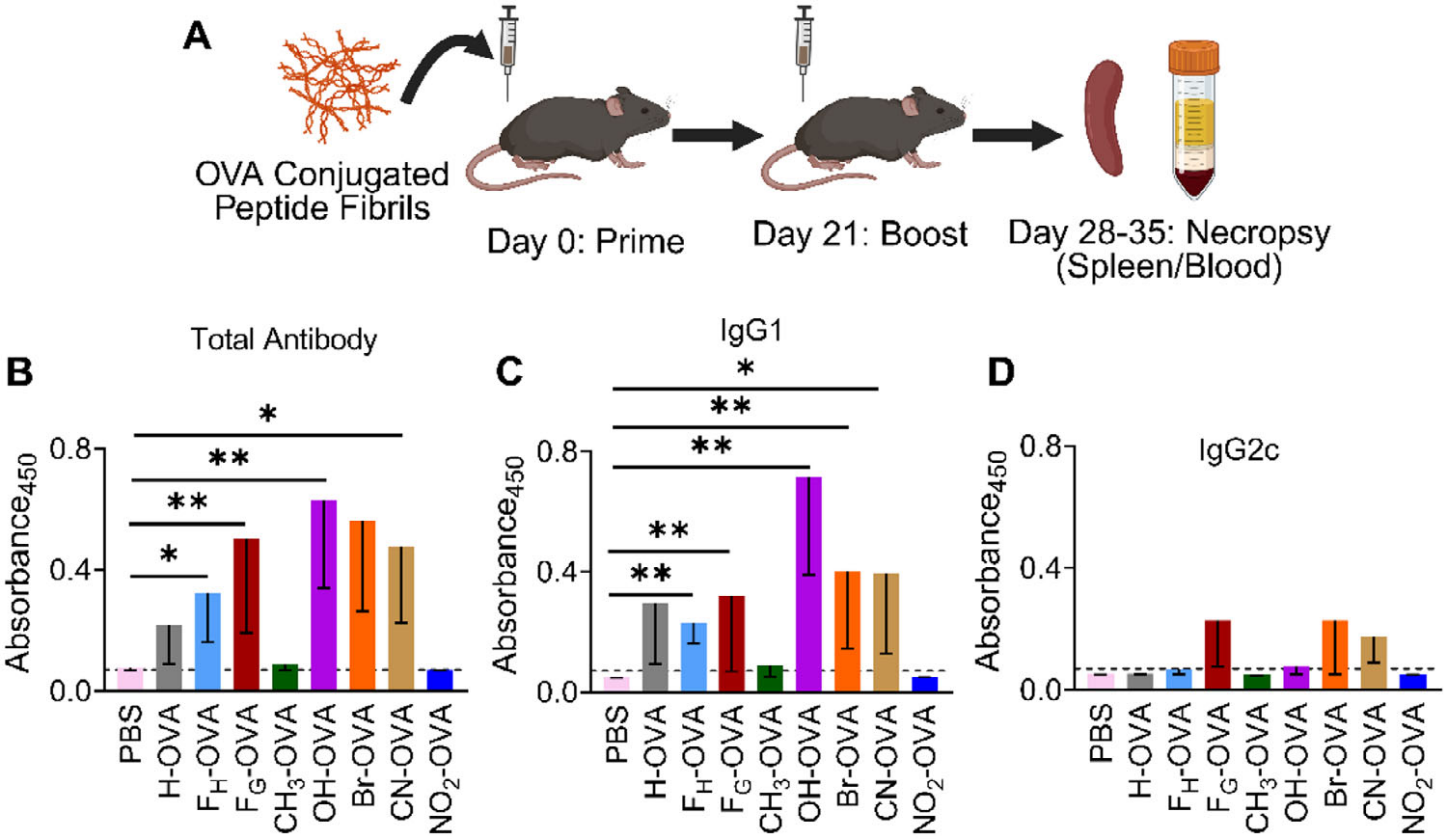
Antibody production upon KFE5 nanofiber vaccination. (A) Mice were primed with a subcutaneous dose of 100 µL of 2 mM peptide nanofibers and then given a subcutaneous boost of 2 mM PNFs 3 weeks later. At 1–2 weeks post‐boost, blood sera were analyzed for antibody production (n = 6). (B) Total antibody production in vaccinated mice along with (C) IgG1 and (D) IgG2c isotypes. *p < 0.05, **p < 0.01, ***p < 0.001, ****p < 0.0001 (n = 6), as determined by a two‐tailed T‐Test with 95% confidence interval, between different substitutions in KFE5 compared to PBS‐treated mice.

To assess antibody responses, we primed and boosted groups of C57BL/6 mice with the OVA‐bearing KFE5 variants and assayed the levels of anti‐OVA antibodies in the sera (Figure 8A). Data indicated that, similar to KFE8, KFE5 acts as an immune adjuvant, leading to the production of anti‐OVA antibodies (Figure 8B). Antibody responses were higher in mice vaccinated with OVA‐bearing F_H_ and F_G_ variants, and this data agrees with our in vitro antigen presentation assays (Figure 7). A stark contrast in immunogenicity was observed between EDGs with an OH substitution and those with a CH_3_ group, with robust antibody production observed with the former and complete abrogation with the latter (Figure 8B). A similar effect was observed with the EWGs, where NO_2_‐KFE5 fibrils bearing OVA were not immunogenic compared to Br or CN‐substituted fibrils that resulted in robust antibody responses (Figure 8B). Analysis of the antibody isotypes revealed that, similar to KFE8, KFE5, and F_H_ peptides, these induced a Th2‐biased response with IgG1 but no detectable IgG2c. In contrast, IgG1 and IgG2c were detected in the sera of mice vaccinated with the OVA‐F_G_ variant, indicating a Th1/Th2 balanced response (Figure 8C). Similar to KFE5, OH modification resulted in IgG1 production alone, whereas brominated and CN‐substituted fibrils induced both IgG1 and IgG2c antibody isotypes (Figure 8C). We next analyzed the Tetramer^+^ CD4^+^T cell populations following splenic antigen recall in vaccinated mice. Data show the presence of antigen‐specific CD4^+^ T cells in almost all groups. A higher percentage of cells was detected in mice immunized with F_G_‐OVA fibrils compared to other groups or controls (Figure S29). These findings are exciting because they show that substituents can not only influence the strength of the immune response but also its composition, opening the possibility of tuning the immune response to the desired correlations of protection.

### 2.10 Transcriptomic Sequencing Analysis

**FIGURE 9:**
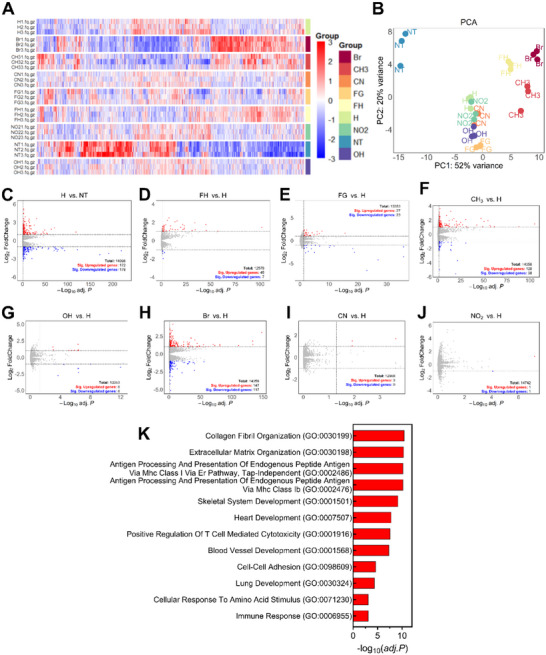
Transcriptomic sequencing analysis of mouse BMDCs treated with various kinds of non‐natural amino acid peptides. (A) Heatmap showing the normalized counts of all differentially expressed (*adj. p* < 0.05) gene clusters of each peptide group compared with the KFE5 peptide. Each count in the gene column was scaled using Z‐score across all samples. (B) Principal Component Analysis (PCA) of mRNA expression variations in BMDCs treated with different peptides. (C–J) Volcano plots comparing gene expression levels between each modified peptide group to the parent peptide. Genes with significantly high expressions are shown in red (*adj. p* < 0.05 and log_2_ fold change > 1), while genes with significantly low expression (*adj. p* < 0.05 and log_2_ fold change < −1) are shown in blue. Gray denotes genes that do not exhibit significant differences (*n* = 3). (K) GO enrichment of unregulated DEGs after KFE5 peptide treatment.

To investigate how structural modifications of FKFEF peptides impact the transcriptomic profiles of BMDCs, we performed RNA sequencing on cells treated with various peptide constructs as shown in Figure 9A,B. Samples within each treatment group clustered tightly, indicating high intra‐group consistency. All peptide‐treated groups were distinctly separated from the non‐treated (NT) group along PC1. Constructs modified with CN, NO_2_, and OH groups closely overlapped with FKFEF, whereas those with Br and CH_3_ substitutions were more distinct from FKFEF along PC1. Additionally, the separation along PC2 for (F_G_, H, and F_H_) suggested that changing the residual chain length contributed to further transcriptomic divergence not captured by PC1. These trends were corroborated by heatmap (Figure 9A) and volcano plots (Figure 9C–J), which highlighted consistent patterns of gene regulation. The FKFEF peptide significantly upregulated 172 genes and downregulated 179 genes. Further functionally annotating these differentially expressed genes (DEG) with Gene Ontology (GO) and Kyoto Encyclopedia of Genes and Genomes (KEGG), we found that upregulated DEGs stimulated by the FKFEF peptide were significantly enriched in biological processes such as the collagen fibril organization, antigen processing and presentation via MHC class I pathway, and positive regulation of T cell‐mediated cytotoxicity (Figure 9K), as well as natural killer cell mediated cytotoxicity pathways (Supplementary Table). Compared to the KFE5(H) peptide, the Br‐substituted variant induced DEGs associated with positive regulations of ERK1 and ERK2 cascade, gene expression, and cell migration. In contrast, the downregulated DEGs associated with CH_3_ and F_G_ modifications were involved in the IL‐17 and TNF signaling pathways. The unregulated DEGs from F_H_ modification also increased the positive regulations of gene expression and MAP kinase activity (Supplementary Table).

## 3 Discussion

The KFE class (FKFE repeats) of amphipathic peptides is a versatile platform for fabricating self‐adjuvanting supramolecular nanofibers for vaccine and immunotherapy applications [7, 48, 49, 50, 51]. Extensive research shows that immune responses to peptide nanofibers can be tuned by altering charge, length, chirality, hydrophobicity, and secondary structure through chemical modifications or the use of non‐natural amino acids [49, 52, 53, 54]. This study investigates how specific changes to the KFE sequence and aromatic groups affect fibril structure and immune function. Changing from KFE8 to KFE5 alters fibril morphology from narrow nanofibrils (∼8–10 nm) to wider, tape‐like structures (50–100 nm). This reorganization results from altered fibrillar packing constraints and fiber growth modes. Structurally, this shift is due to changes in phenylalanine stacking at the ends: in KFE8, out‐of‐register terminal phenylalanine stabilizes tight fibrils through π–π interactions, promoting linear growth and restricting lateral expansion. Phenylalanine residues also arrange at fiber edges, forming hydrophobic strips that act as bridges and lower barriers for lateral merging. These edge interactions nucleate fiber associations, leading to wider tapes and bundling. Overall, the transition from core‐dense nanofibrils to edge‐dominated tapes reflects a shift from isolated cores to edge‐mediated networks.

Beyond backbone length, aromatic substituents' electronic and steric properties strongly influence fibril chemistry and morphology. EWGs like CN and NO_2_ reduce π–π stacking, producing thinner, more planar fibrils with weaker inter‐ring interactions [55], whereas Br, despite being an EWG, results in broader, denser tapes, showing that sterics and complex electronic effects modify stacking beyond simple electron withdrawal [56, 57]. These differences highlight the role of substituent‐specific sterics and electronics in assembly pathways, with minor changes significantly shifting intra‐ and inter‐fibril interactions. EDGs such as OH essentially inhibit fibrillization, likely via hydrogen bonding or disrupting stacking, while methyl (CH_3_) promotes fibril formation but induces twisting due to increased conformational strain [58, 59]. This emphasizes the delicate balance of hydrophobic, steric, and stacking interactions for stable β‐sheet assembly. Modifications of benzyl group length, like shorter F_G_ or longer F_H_ variants, also affect fibril dimensions: F_G_ forms shorter, thinner fibrils, while F_H_ promotes bundled, thicker fibers. Overall, substituents shape fibril width, thickness, and organization through electronic and steric effects [60, 61, 62].

Spectroscopic differences between the variants also reveal nuances in packing and orientation. CD spectra show variations reflecting changes in β‐sheet geometry and alignment. The OH‐substituted peptide shows a strong CD signal near 235 nm despite no fibrils being observed by TEM, indicating aromatic stacking can occur without fibril formation or in other ordered states [63]. ThT assays confirm β‐sheet‐rich structures, highest in Br and CN variants, with fibrils seen in TEM. EDG‐containing peptides generally produce weaker ThT responses, suggesting less robust fibril formation or altered β‐sheet organization less favorable to ThT binding [64]. FT‐IR corroborates β‐sheet dominance, with subtle shifts indicating orientation differences; for instance, the F_H_ variant tends toward parallel β‐sheets, as also observed in the CD spectra obtained for this peptide [65]. These markers reveal interactions between aromatic stacking, edge dynamics, and fibril structure. The ability to form a self‐supporting gel depends on fibril network connectivity rather than fibrillization itself. The parent KFE5 peptide does not gel at 5 mM, indicating that even well‐formed isolated fibers may not assemble into a continuous network without sufficient inter‐fibril interactions. The addition of EWGs restores gelation across all conditions, showing that improved inter‐fibril contacts, possibly through increased edge hydrophobic interactions or favorable stacking at junctions, are crucial for network formation. Conversely, EDG substitutions hinder gelation, underscoring that firm gels require a carefully balanced combination of fibril density, contact geometry, and surface chemistry to support a connected network rather than isolated fibrils. The ability to tune mechanical properties through specific substituent chemistry offers a way to customize the material's physical environment [34, 66, 67] for desired immunological outcomes, whether it's prolonged antigen exposure, specific drainage features, or controlled release behavior.

One key finding from the data is that the chemical composition of fibrils impacts both innate and adaptive immune responses. When primary DCs are exposed to peptide fibrils containing EWGs, they mount a pro‐inflammatory response. In contrast, DCs treated with fibrils containing EDGs mount a weaker response. Similarly, the fibril variant with F_H_ induces a strong pro‐inflammatory environment characterized by cytokine release, whereas the F_G_ variant elicits a weaker response. These patterns suggest that the density and chemical makeup of fibrils play a role in activating DCs and influencing their subsequent processing of antigens [68, 69]. Dimensions of fibrils also influence how antigens are processed and presented. Our results indicate that fibril length, rather than diameter, is a key determinant of cellular uptake and antigen presentation. Peptide variants forming shorter fibrils, including OVA‐conjugated F_H_, F_G_, and CN, elicited higher IL‐2 responses and were internalized via clathrin‐mediated endocytosis, as evidenced by their sensitivity to CPZ treatment. In contrast, longer fibrils exhibited reduced antigen presentation and relied predominantly on dynamin‐ or actin‐dependent uptake pathways, showing marked sensitivity to dynasore or cytochalasin A inhibition. Consistent with prior reports demonstrating length‐dependent control of antigen cross‐presentation in supramolecular peptide nanofibers, these findings suggest that reduced fibril length promotes more efficient, clathrin‐mediated cellular entry and antigen processing, thereby enhancing downstream T‐cell responses. This observation aligns with prior work from the Collier lab, which demonstrated that shortening supramolecular peptide nanofibers via structure‐modifying peptides improves antigen cross‐presentation and T‐cell activation [70].

In this context, the connection between fibril structure and antigen presentation supports an efficiency theory: when antigens are more accessible or quickly internalized, it leads to stronger T cell responses. Antibody responses also vary depending on the identity of the substituent. Among EDGs, CH_3_ doesn't trigger anti‐OVA antibodies, whereas OH elicits a Th2‐dominated response with strong IgG1 production. For EWGs, CN and Br produce balanced Th1/Th2 antibody profiles (detectable IgG1 and IgG2c), whereas NO_2_ is associated with reduced antibody production but maintains some CD4^+^ T cell activity, suggesting a disconnect between humoral and cellular responses. These isotype patterns are instructive because IgG1 and IgG2c are associated with Th2‐type and Th1‐type immune polarizations [71], respectively, with implications for deploying these materials against different pathogens or in various vaccination strategies. To assess fibril stability, we measured ThT fluorescence over 7 days at neutral pH and room temperature. Data indicated no loss of ThT fluorescence. Increased fluorescence was detected for some variants, presumably due to lateral aggregation (Figure S30A). Proteolytic stability was also assessed by incubating the fibrils with trypsin alone or with a trypsin and chymotrypsin cocktail [72, 73]. Under both conditions, most peptide fibrils exhibited substantial degradation, with the NO_2_ variant emerging as a notable exception, showing no detectable degradation (Figure S30B,C). Despite its enhanced proteolytic stability, the NO_2_ variant elicited low IL‐2 responses, indicating that antigen persistence alone does not account for the observed differences in immune responses. While these studies are not directly indicative of in vivo stability or persistence, they provide valuable insights into the fibrils' stability and degradation patterns of the fibrils under controlled conditions.

RNA‐seq and transcriptomics deepen understanding of fibril design's immunomodulatory effects. Peptides with CN, NO_2_, and OH closely mimic KFE5‐like gene expression, maintaining a core signature, while Br and CH_3_ induce distinct transcriptional profiles, affecting ERK signaling and inflammation. Backbone length also influences gene networks, where F_H_ fibrils impact additional pathways compared to F_G_ fibrils. Ultimately, DC transcriptional programs demonstrate these materials actively rewire immune networks, enabling tailored therapeutic effects. From a practical design standpoint, these findings offer a useful framework. Selecting substitutions that produce desired fibril density and surface chemistry allows fine‐tuning of immune activation and balancing responses, leading to vaccine platforms with customized adjuvanticity. For example, achieving a balanced Th1/Th2 profile benefits vaccines requiring strong antibody responses and cellular immunity. Conversely, variants reducing inflammatory signaling while preserving antigen presentation can help minimize autoimmune risks or promote tolerogenic outcomes.

This work shows that two design axes influence KFE‐based fibrils' immunomodulatory performance: (i) peptide backbone length, affecting core packing and lateral growth, and (ii) aromatic substituent identity, which modulates inter‐fibril contacts, β‐sheet orientation, and surface chemistry. This work also demonstrates that designed peptides like KFE5 fibrillize instantaneously, without a significant lag phase, unlike pathological amyloids. This explains why hydrogels fabricated from designed amphiphatic peptides such as KFE8, MAX1, or RADA16 exhibit minimal cytotoxicity in cell culture and animal models [74, 75, 76]. The link between nanoscale morphology, mechanics, and immune signaling indicates that precise molecular changes in aromatic residues and sequence length lead to predictable shifts in fibril structure, material properties, and immunity. However, the connections between specific surface chemistries and DC signaling remain unclear. Further study of receptor engagement and antigen‐processing would clarify how fibril chemistry affects immunity. Additionally, examining how edge‐driven nucleation influences assembly and network formation could inform scalable manufacturing of peptide nanofiber vaccines.

## 4 Conclusion

In summary, our findings show that chemical substituents on KFE5 nanofibers effectively control their immunological and structural features. Altering side‐chain chemistry can enhance fibril morphology, modulate innate immune activation, and steer adaptive immune responses. This work advances the targeted design of peptide‐based nanomaterials for applications such as immunotherapy, vaccines, and other biomedical fields, highlighting the crucial influence of the chemical environment on biological effects.

## 5 Methods

### 5.1 Peptide Synthesis

Standard Fmoc‐SPPS chemistry was used to synthesize the peptides on Rink Amide resin. Oxyma [ethyl 2‐cyano‐2‐(hydroxyimino)acetate] and *N,N′*‐Diisopropylcarbodiimide (DIC) were used as coupling agents on a Liberty Blue microwave‐assisted synthesizer (CS Bio, CA). Peptide cleavage was carried out in a cocktail of trifluoroacetic acid (TFA), triisopropylsilane (TIS), and H_2_O (95:2.5:2.5) and diethyl ether was used for extraction. The resulting pellet was frozen and lyophilized in acetonitrile/water (ACN/H_2_O) mixture (50:50). Peptides were purified (∼90%) using High Performance Liquid Chromatography (HPLC) on a Dionex Ultimate 3000 HPLC (Agilent Zorbax SB‐C18, 21.2 × 150 mm, 5 µm) equipped with a diode‐array detector. ACN/H_2_O gradient was applied to an Agilent Poroshell RP‐C18 column (4.6 mm × 150 mm), operating at a 1 mL/min flow rate. MALDI‐TOF mass analysis (Shimadzu MALDI‐8030) was used to confirm peptide identity using α‐cyno‐4‐hydroxycinnamic acid matrix (Bruker Daltonics, MA) (Figures S1–S8). OVA_323–339_ conjugated peptides were purchased from GenScript (GenScript USA Inc.) or P3 Bio‐Systems (Louisville, KY) and used without further modification.

### 5.2 Peptide Fibrillization

Peptide self‐assembly was achieved by dissolving the purified peptide in Ultrapure Water (UPW) to prepare 1 mM stocks for further dilution and use. OVA‐conjugated peptides were first dissolved in UPW, then diluted in 10× PBS (phosphate‐buffered saline) to a final concentration of 1 mM in 1× PBS for immunizations. The pH of each peptide solution was measured before in vitro and in vivo studies (Table S5). Notably, the pH of UPW was ∼5.0. Consistently, upon dissolving the non‐OVA peptide variants in water, the pH of the resulting solutions remained at ∼5.0. In contrast, for the OVA‐conjugated variants, the pH decreased to ∼3.0 immediately after dissolution in water. Upon addition of 10× PBS to achieve a final concentration of 1× PBS, the pH of these solutions increased to 7.0. Since all cell‐based experiments were performed using fibril solutions prepared in PBS, the pH was maintained at physiological pH (7.0) throughout these studies.

### 5.3 Peptide Modeling and Molecular Dynamics

The FKFEF peptide, excluding the peptide modifications, was used in AlphaFold3 with 30 copies. The most complete and continuous model was extended to a full 16‐strand sheet of the predicted 2‐start fiber with edge contacts between the two parallel fibers using COOT and PyMol. Each fiber was assembled similarly to that in KFE8 [36], with the phenylalanine residues forming a hydrophobic core sandwiched between the peptide β‐sheet forming backbone. This protofiber model, with the N‐acetyl and amidated C‐terminus modifications added, was allowed to equilibrate in explicit TIP3 solvent with 150 mM NaCl, using NAMD3 with initial annealing, with anti‐parallel β‐sheet hydrogen binding restraints applied using the extra bonds option, followed by a 500 ns full free MD simulation. The resulting protofilament model was then extended to a 48‐strand sheet, 2‐start fiber, dual parallel fiber bundle of 192 KFE5 molecules. This extended model was minimized and annealed with model anti‐parallel β‐sheet hydrogen bonding restraints as for the protofilament model. The 192‐chain model was then equilibrated for 50 ns at 300 K. The same procedure was then used to prepare a 348 peptide, 4‐fiber, 48‐peptide sheet, 2‐start fiber, model for a 100 ns restraint‐free molecular dynamics simulation run.

### 5.4 Thioflavin T Assay

For ThT assays, 50 µM fibrillated peptide solutions (diluted in UPW from fibrillated peptide solutions as stated above) were mixed with 20 µM ThT solution, and fluorescence was monitored at 485 nm upon excitation at 440 nm in a 96‐well plate using a Synergy HT plate reader (Biotek, USA). The OVA‐conjugated peptide fibrils were used to assess long‐term stability over a 7‐day period.

### 5.5 Enzyme Degradation Assay

To assess the stability of the OVA‐conjugated peptide fibrils, preformed peptide nanofibrils were incubated with trypsin alone (0.1 µg/µL) or with a trypsin–chymotrypsin cocktail (0.1 µg/µL at 1:1 molar ratio). The degradation kinetics were monitored at defined time intervals using the Thioflavin T (ThT) assay, as described above.

### 5.6 Transmission Electron Microscopy

Peptide solutions (10 µM; diluted in UPW from fibrillated peptide solutions as stated above) were applied directly to 200‐mesh, carbon‐coated copper grids for 2 min and stained with 1% uranyl formate for 1 min. Excess stain and the peptide solution were blotted using filter paper, and grids were washed thrice with UPW. Darkfield images were acquired on a JEOL (Akishima, Japan) JEM‐2100F Field‐Emission STEM microscope at an accelerating voltage of 120 kV.

### 5.7 Width and Length Measurements of Fibrils

FIJI software was utilized to measure the widths of the fibers, and the measurement scale was established using the scaling factor displayed in each image. Individual fibers were selected for measurement, avoiding aggregates. The width of each fiber was measured using the line tool by drawing a line across the fiber's width at an angle approximately perpendicular (90°) to its orientation to ensure accuracy. Similar measurements were done to calculate the length of the fibrils. The start and end points of the line were determined based on the contrast between the fiber and the surrounding background. Approximately 30 fibers (∼100 measurements) were randomly taken for each sample.

### 5.8 Fourier Transform Infrared Spectroscopy

FT‐IR measurements for secondary structural analysis were conducted using 1 mM peptide solutions (UPW) with a Bruker (Billerica, MA) Alpha II FT‐IR instrument equipped with a Smart Performer single‐reflection ATR accessory and an Au crystal sample stage. The background and buffer spectrum were collected and subtracted from the sample using OPUS software. An average of 24 scans for each peptide was employed for each sample measurement. Data were analyzed using GRAMS/AI software (Thermo Scientific, USA). Second derivative spectra were calculated from the absorbance spectra in the Amide I region using a Savitzky‐Golay filter, third order, with a nine‐point window. The second derivative spectra analyzed between 1610 cm^−1^ and 1710 cm^−1^ were fit with six or seven Gaussian curves, informed by the Akaike information criterion, and the peak positions were compared to literature reports [77, 78, 79].

### 5.9 Circular Dichroism Spectroscopy

CD spectra were recorded using 100 µM peptide solutions (diluted in UPW from fibrillated peptide solutions as stated above) on a Jasco (Hachioji‐shi, Japan) J‐815 CD spectrometer (3 samples each) at RT (25°C). The spectra (average of three scans for each sample) were collected using the following parameters: wavelength 190–260 nm, bandwidth of 1 nm, data pitch of 0.5 nm step. The solvent background was subtracted from each spectrum. BeSTSel software was used for decnvulating the spectra obtained for different peptide variants [80].

### 5.10 Mechanical Properties of Peptides

An ARES 2000ex rotational rheometer (TA Instruments, New Castle, DE) was used to evaluate the viscosity and viscoelastic properties of the peptides. Peptides were freshly solubilized in deionized water at 5 mM and allowed to incubate for 10 min to allow for complete solubilization. Next, 170 µL of each peptide solution was pipetted directly onto the rheometer stage. A 20 mm parallel plate geometry was lowered to a gap of 200 µm. Storage modulus (G′) and loss modulus (G″) were measured as a function of strain amplitude of 0.05–50% at an angular frequency of 10 rad/s to determine the linear viscoelastic region. Next, G′ and G″ were measured as a function of angular frequency (1–10 rad/s) at a strain of 1%. Last, the viscosity of each peptide solution was measured as a function of shear rate (0.1–100 s^−1^) to determine if samples exhibited non‐Newtonian behavior. Separately, a Hagen‐Poiseuille viscometer (microVISC, Rheosense, San Ramon, CA) was used at a shear rate of 1000 s^−1^ to validate measured viscosity trends.

### 5.11 X‐ray Scattering Studies

SAXS data of the KFE5 X‐phenyl substituted peptide solutions were collected using a Rigaku (Woodlands, TX) BioSAXS‐1000 camera on an FRE^++^ X‐ray generator with an ASC‐96 Automated Sample Changer held at 10°C. A matching buffer was collected for each sample. The detector was calibrated using a silver behenate powder sample, following the manufacturer's recommended procedure. The SAXS samples (KFE5 and its variants) were prepared by dissolving 2–3 mg of lyophilized peptide in 400 µL of deionized water to produce a final concentration of 4 mg/mL. The samples were then vortexed, and precipitants were removed by centrifugation at 13.3 rpm for 5 min. Although the samples formed gels, these remained fluid under hydrostatic pressure, permitting pipetting by the ASC‐96 liquid handling system. The cyano variant of KFE5 was an exception. It remained a gel at 4 mg/mL and needed to be diluted to 2 mg/mL to be pipetted or flowed in the ASC‐96 liquid sample handling system. Processing was performed in SAXSLab (Rigaku) and SAXNS‐ES (https://xray.utmb.edu/SAXNS). Analyses were performed in Primus/GNOM [81], BIFT [82, 83], and gnuplot (http://www.gnuplot.info).

WAXS samples were prepared by dissolving 3–5 mg of lyophilized peptide in 500 µL of methanol. Each sample was pipetted into MiTeGen capillary in 30 µL aliquots, left to dry, and additional aliquots added until the entire sample was transferred into the capillary. Powder diffraction data were collected on a Rigaku R‐AXIS‐IV^++^, with a Rigaku FRE^++^ Cu X‐ray source using four 30‐min frames. The diffraction images were processed using Fit2D and calibrated with a sucrose sample. The curves from multiple frames were averaged in Primus. In addition to the strong powder diffraction, the data contained broad background scattering from the capillary and the amorphous gel. This background was removed using d1Dplot and the resulting powder diffraction peaks fit. X‐ray diffraction peaks were each fit to a simple Gaussian with a common theta‐dependent peak width σ using gnuplot.

### 5.12 FRET Biosensor Assays

HEK293T biosensor cells (HEK293T‐α‐syn‐CFP/α‐syn‐YFP) were obtained from David Holtzman [84]. DMEM (Dulbecco's modified high glucose Eagle's medium), supplemented with 10% FBS (fetal bovine Serum), and 1% PenStrep (penicillin/streptomycin) was used for growing the cells, which were subsequently plated in 96‐well plates at a density of 3.5 × 10^4^ cells/well. Briefly, the peptide nano‐fibers (50 nM and 500 nM) were applied post 24 h of cell plating. Before applying, fibrils of KFE5 and its variants, along with α‐Syn pre‐formed fibrils (50 nM) were diluted in fibrillization buffer to the appropriate concentration and sonicated in a cup horn bath sonicator for 3 min, mixed with Lipofectamine 3000 (Invitrogen) at 0.5 µL Lipofectamine per well, incubated for 10 min at room temperature, and then applied to the biosensor cells. Post 48 h, the cells were trypsinized, transferred to a separate 96‐well plate, and fixed with the help of 4% paraformaldehyde for 15 min at 4°C in the dark [84]. Cells were resuspended in 150 µL chilled FACS buffer and analyzed on a Beckman Coulter CytoFLEX S, according to a previously established protocol [7]. The data was analyzed using FlowJo software. Images were collected on a Lionheart FX Automated microscope (BioTek Instruments, Inc.).

### 5.13 Bone Marrow‐Derived Dendritic Cells Culture

The femurs and tibiae of mice of C57BL/6 mice were flushed, and single‐cell suspensions were prepared, following removal of red blood cells (RBCs) using lysis buffer (Invitrogen, USA). After washing twice using HBSS (Hanks balanced salt solution), cells were differentiated for 7–9 days in complete Roswell Park Memorial Institute (RPMI) 1640 media containing heat‐inactivated Fetal bovine serum (FBS) (10%), β‐mercaptoethanol (55 µM), sodium pyruvate (1 mM), 10 mM 4‐hydroxyethylpiperazine ethane sulfonic acid (HEPES), 1× MEM (Minimum Essential Medium) non‐essential amino acids, 20 ng/mL granulocyte‐macrophage colony‐stimulating factor (GM‐CSF), 5 ng/mL IL‐4 and 100 µg/mL Penicillin‐Streptomycin (pen‐strep) in 150 mm dish (25 mL/dish) in an incubator at standard conditions (37°C and 5% CO_2_). Cells were harvested using gentle washing with MEM media between days 7–9 for experiments.

### 5.14 Antigen Presentation Assays

To assess antigen presentation, DCs (5 × 10^4^ cells/well) were plated in 96‐well plates and treated with OVA‐conjugated peptides at various concentrations (0.1, 1, 2.5, 5, 10 µM) for 24 h. The cells were then washed with PBS to remove extracellular fibrils. The T‐cell hybridoma cell line DOBW (kind gift from Dr. Clifford V. Harding, Case Western Reserve University, Cleveland, OH, USA) [85] were overlaid (1:5, DC: hybridoma) for 16 h. The supernatant was collected, and IL‐2 levels were quantified by ELISA (Biotechne, Cat#: DY402‐05). Briefly, 96‐well high‐binding ELISA plates (CORNING Costar 3361) were coated with 100 µL of the capture antibody in 1× PBS (pH 7.4) overnight at 4°C and then blocked with blocking buffer (1% BSA in 1× PBS, pH 7.4) for 1 h. After the blocking step, cell culture supernatants or standards were added to the wells in a diluent buffer (0.1% BSA and 1× TBST) and incubated for 2 h, followed by the addition of a detection antibody (100 µL/well in the diluent buffer for 2 h). Finally, streptavidin‐HRP (Horseradish peroxidase) (100 µL/well) in diluent buffer was added for 20 min, followed by 100 µL of TMB (Tetramethylbenzidine) substrate. The reaction was stopped using 50 µL of 1 M phosphoric acid, and absorbance was measured at 450 nm. The plate was washed 3× using 300 µL/well of wash buffer between each incubation step. The amount of IL‐2 produced was measured from the standard curve.

To evaluate the effect of endocytic inhibitors on coacervate uptake, BMDCs (5 × 10^4^ cells mL^−^^1^) were seeded in 96‐well plates and pretreated for 0.5 h with chlorpromazine (CPZ, 5 µg/ml), IPA3 (10 µM), Dynasore (Dyn, 80 µM), Cytochalasin A (CytoA, 10 µM), and Rottlerin (Rot, 10 µM). OVA‐conjugated peptide fibrils were then added, and after 24 h of incubation, the cells were washed and co‐cultured with DOBW cells for 16 h. IL‐2 production was subsequently quantified by ELISA as described above.

### 5.15 Immunizations and Antibody Titers

All experiments were approved by the Institutional Animal Care and Use Committee (IACUC) at WashU. Female C57BL/6 mice (Jackson Labs 4–6 weeks old) were housed under standard conditions. Mice (n  =  6/group) were subcutaneously (s.c.) vaccinated (100 µL of 2 mM solution) and boosted 3 weeks later with the same dose. Control mice received saline. 7–10 days post‐boost, blood was collected using cardiac puncture, and serum was extracted by centrifugation. High‐binding ELISA plates (Corning #3361) were coated with 20 µg/mL OVA peptide in PBS and incubated overnight at 4°C and subsequently blocked for 1 h at RT with 1× ELISA diluent (Invitrogen; 100 µL/well). After blocking, 50‐fold dilutions of the sera were prepared in ELISA diluent and added to the wells (100 µL/well for 2 h at 25°C) followed by a secondary HRP‐conjugated goat anti‐mouse IgG (1:5000, 100 µL/well) for 30 min. For isotyping, HRP‐conjugated isotypes (IgG1, IgG2c; Southern Biotech) were added (1:4000, 100 µL/well for 30 min at 25°C) after the addition of sera prior to development. Plates were developed using TMB substrate (100 µL/well) for 15 min, and the reaction was quenched with 1 M phosphoric acid (50 µL/well). The plate was washed 3× using 300 µL/well of wash buffer between each incubation step. Absorbance was recorded at 450 nm using a BioTek Synergy microplate reader, and background absorbance from non‐antigen‐coated wells was subtracted.

### 5.16 Cellular Immune Responses Through Flow Cytometry Analysis

Following euthanasia, spleens were dissociated using syringe plungers through 100 µm filters and washed with HBSS (Hank's Balanced Salt Solution) containing 5% FBS. Cell pellets were resuspended in 2 mL RBC lysis buffer for 2 min, washed with HBSS, and resuspended in complete RPMI‐1640. Cells were counted and plated in triplicate (10^6^ cells/well in 250 µL), and antigen‐specific T cell populations were recalled by incubating with RPMI‐1640 media containing 0.2 mg/mL of the cognate antigen OVA. After 96 h, cells were washed twice with 200 µL PBS, then resuspended in 100 µL PBS containing eFluor506 (eBioscience, 65‐0866‐14) and Fc‐Block (BioLegend, 101302) for 30 min. Cells were then washed 2× with FACS (Fluorescence‐activated cell sorting) wash buffer (FWB, 1× PBS + 10% FBS) and stained with 50 µL FWB with OVA‐specific Tetramer (I‐A^b^ PE, MBL TS‐M710‐1), 50 µL of anti‐CD3 (BV421, Biolegend 100228), and 50 µL of anti‐CD4 (PerCP‐Cy5.5, eBioscience, 45‐0042‐82) for 30 min. Cells were washed 2× with FWB and resuspended in 4% paraformaldehyde for fixation. After 15 min, cells were washed three times and resuspended in 200 µL FWB for acquisition. Cells were analyzed on a Beckman Coulter CytoFLEX S, and data were analyzed using FlowJo software. Single‐color control beads were used for compensation. For analysis, cells were gated on single cells, live, CD3^+^, CD4^+,^ and Tetramer^+^ populations.

### 5.17 RNA Sequencing

Mature BMDCs (5 × 10^5^ cells/well) were plated in 24‐well plates and treated with 10 µM peptide solutions for 24 h, following which, the QIAGEN RNeasy Mini Kit (Cat#: 74104) was used for the isolation and purification of RNA from the treated DCs. Briefly, 350 µL of Buffer RLT (Qiagen, Mississauga, Canada) was added to the pelleted cells and vortexed for complete homogenization. It was then followed by an addition of 350 µL of 70% lysate and mixed well by pipetting. The entire volume of the sample was then transferred to a RNeasy Mini spin column placed in a 2 mL collection tube. The tube was centrifuged for 20 s at 8000× g at 4°C. The flow‐through was discarded and 700 µL of Buffer RW1 was added, following a 20 s 8000 × g centrifugation at 4°C. Similarly, after discarding the flow‐through, the spin column was centrifuged under similar conditions by the addition of 500 µL of Buffer RPE 2×. The RNA spin column was placed in a new 1.5 mL collection tube, followed by the addition of 50 µL of RNase‐free water. The solution was centrifuged at above mentioned conditions, and the RNA yield was estimated for each sample using the Nucleic Acid Quantification Protocol in a BioTek Synergy H1 Microplate Reader (Agilent USA).

The mRNA library construction, quantification, and sequencing via Illumina platforms were performed by Novogen (Sacramento, CA, USA) with stepwise quality control checks, which include read counts normalization, model‐dependent p‐value estimation, and False Discovery Rate (FDR) value estimation based on multiple hypothesis testing. This is preceded by filtering raw reads, mapping clean reads to a reference genome using HISAT2, and determining Fragments Per Kilobase per Million mapped Fragments (FPKM) values for all samples. Differentially expressed genes were evaluated based on their log_2_ fold change and adjusted p‐values. Those with |log_2_(fold change)|>0 and adjusted p‐values < 0.05 were considered to be differentially expressed and significant.

### 5.18 Transcriptome Sequencing Analysis

Data processing and visualization were performed as previously mentioned [86]. Briefly, raw data were uploaded to the Galaxy website (http://www.usegalaxy.org), quality checked (FastQC, Galaxy ver. 0.74) and mapped to the Mus musculus Genome Reference Consortium Mouse Build 39 (RNA Star, Galaxy ver. 2.7.11b). FeatureCount (Galaxy ver. 2.0.3) was used to count the number of reads per annotated gene in each mapped BAM file. Raw counts of genes were transferred to RStudio (ver. 2024.12.1) and normalized with the DESeq2 package (ver. 1.42.1). The heatmap was created with the heatmap package (ver. 1.0.12), and principal component analysis (PCA) plot and volcano plot were generated using the ggplot2 package (ver. 3.5.2). Functional annotation clustering using Gene Ontology (GO) and pathway analysis of DEGs with the Kyoto Encyclopedia of Genes and Genomes (KEGG) were conducted through the Database for Annotation, Visualization, and Integrated Discovery (DAVID) v2023q4 [87].

### 5.19 Statistical Analysis

Statistical analysis was performed in GraphPad Prism. Data are expressed as mean ± S.E.M. Statistical analysis was performed using a one‐way or two‐way ANOVA with Tukey/Sidak's/Dunnett's multiple comparison test. *p ≤ 0.05, **p ≤ 0.01, ***p ≤ 0.001, ****p ≤ 0.0001.

## Conflicts of Interest

The authors declare no conflict of interest.

## Supporting information




**Supporting File**: advs73940‐sup‐0001‐SuppMat.docx.

## Data Availability

The data that support the findings of this study are available from the corresponding author upon reasonable request.
