# Supplementary material for: Substituent‐Based Modulation of Self‐Assembly and Immunogenicity of Amphipathic Peptides
Source: Adv Sci (Weinh). 2026 Jan 20;13(18):e18567. doi: 10.1002/advs.202518567 (PMC13042944; doi:10.1002/advs.202518567)
Supplement: Supplementary file 1 — Supporting File: advs73940‐sup‐0001‐SuppMat.docx. [file ADVS-13-e18567-s001.docx]

**Supporting Information**

**Substituent-based Modulation of Self-Assembly and Immunogenicity of Amphipathic Peptides**

Anirban Das^a,b,#^, Ushasi Pramanik^a,#^, Elise M. Brown^a^, Chih-Yun Liu^a^, Huan Gong^c^, Jonathan Fascetti^a^, Mark Gibson^a^, Samuel Stealey^d^, Silviya P. Zustiak^d^, Cory Berkland^a,b^, Piyoosh Sharma^a^, Meredith E. Jackrel^b^, Mark A. White^e^, and Jai S. Rudra^a,*^

^a^Department of Biomedical Engineering, Washington University in St. Louis, St. Louis, MO, USA

^b^Department of Chemistry, Washington University in St. Louis, St. Louis, MO, USA

^c^Department of Pharmaceutical Chemistry, The University of Kansas, Lawrence, KS, USA

^d^Department of Biomedical Engineering, Saint Louis University, St. Louis, MO, USA

^e^Sealy Center for Structural Biology and Molecular Biophysics & Department of Biochemistry and Molecular Biology, University of Texas Medical Branch, Galveston, TX, USA

^*^Correspondence: Jai S. Rudra, [srudra22@wustl.edu](mailto:srudra22@wustl.edu)

^#^These authors contributed equally.

**Table of Contents**

**Figure S1**. MALDI-TOF and HPLC spectra of KFE5 S4

**Figure S2**. MALDI-TOF and HPLC spectra of KFE5(F_H_) S5

**Figure S3**. MALDI-TOF and HPLC spectra of KFE5(F_G_) S6

**Figure S4**. MALDI-TOF and HPLC spectra of KFE5(CH_3_) S7

**Figure S5**. MALDI-TOF and HPLC spectra of KFE5(OH) S8

**Figure S6**. MALDI-TOF and HPLC spectra of KFE5(Br) S9

**Figure S7**. MALDI-TOF and HPLC spectra of KFE5(CN) S10

**Figure S8**. MALDI-TOF and HPLC spectra of KFE5(NO_2_) S11

**Figure S9.** TEM images of KFE8 and KFE5 S12

**Figure S10.** A 500 ns MD Simulation of the AlphaFold3 KFE5 model S13

**Figure S11**. TEM images for KFE5 variants S14

**Table S1.** BeSTSel deconvolution analysis for CD spectra of KFE5 peptide variants.........................................................................................................……………………S14

**Figure S12.** Concentration-dependent relative ThT fluorescence for all peptides ... S15

**Figure S13.** FT-IR spectroscopy of KFE5 variants... S16

**Figure S14.** Gel formation, TEM images, and viscosity measurements S17

**Figure S15.** Powder diffraction peak fitting for KFE5 variants S18-S19

**Table S2**. WAXS peaks for KFE5 peptides S20

**Figure S16**. Cytokine and chemokine production by KFE5 variants S21

**Table S3.** Table for Chemokine and Cytokine production by KFE5 variants S21

**Figure S17.** FRET analysis for α-synuclein aggregation by KFE5 variants (0.5 µM) S22

**Table S4.** Sequences for OVA-conjugated KFE5 variants S23

**Table S5.** pH of peptide solutions used in this study…………………………………………..S23

**Figure S18.** MALDI-TOF and HPLC spectra of KFE5-OVA S24

**Figure S19.** MALDI-TOF and HPLC spectra of KFE5(F_H_)-OVA S25

**Figure S20.** MALDI-TOF and HPLC spectra of KFE5(F_G_)-OVA S26

**Figure S21.** MALDI-TOF and HPLC spectra of KFE5(CH_3_)-OVA S27

**Figure S22.** MALDI-TOF and HPLC spectra of KFE5(OH)-OVA S28

**Figure S23.** MALDI-TOF and HPLC spectra of KFE5(Br)-OVA S29

**Figure S24.** MALDI-TOF and HPLC spectra of KFE5(CN)-OVA S30

**Figure S25.** MALDI-TOF and HPLC spectra of KFE5(NO_2_)-OVA S31

**Figure S26.** TEM and CD of OVA-conjugated KFE5 variants S32

**Figure S27.** Diameter and length of OVA-KFE5 variants……………………………………..S33

**Figure S28**. Uptake mechanisms of OVA-KFE5 variants in DCs……………………………..S34

**Figure S29.** Percent OVA-specific CD4^+^T cells S35

**Figure S30.** Enzymatic stability of OVA-KFE5 variants………………………………………S36

**
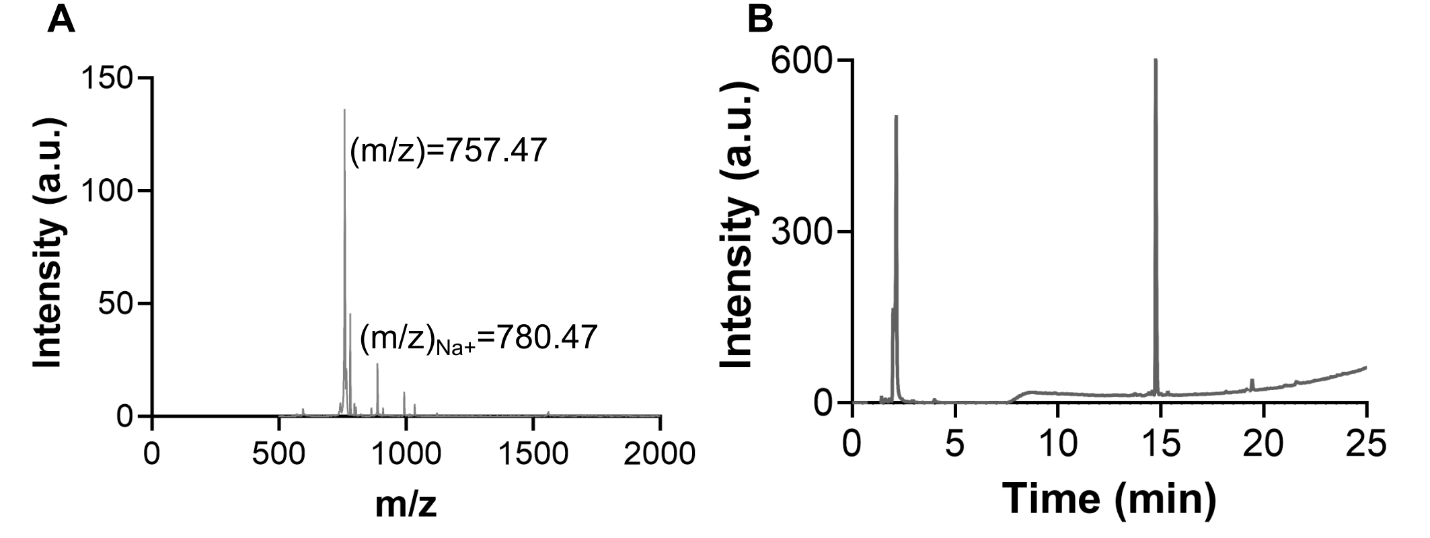
**

**Figure S1.** (A) MALDI-TOF and (B) HPLC profiles of KFE5.


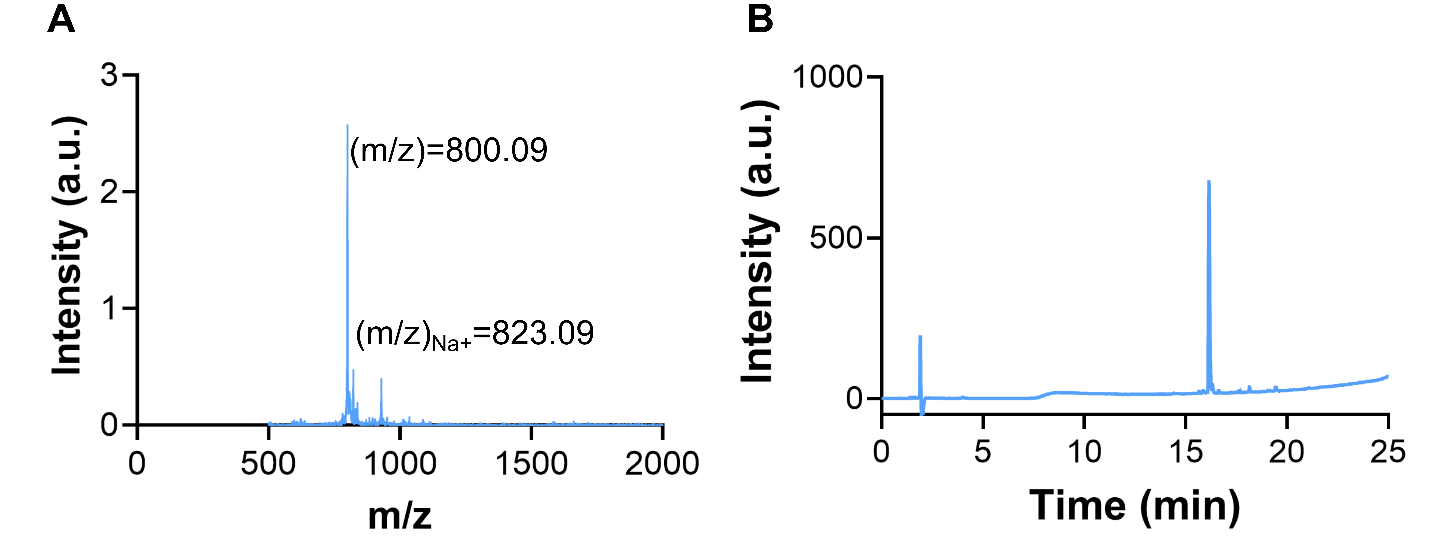


**Figure S2.** Representative (A) MALDI-TOF and (B) HPLC profiles of KFE5(F_H_).


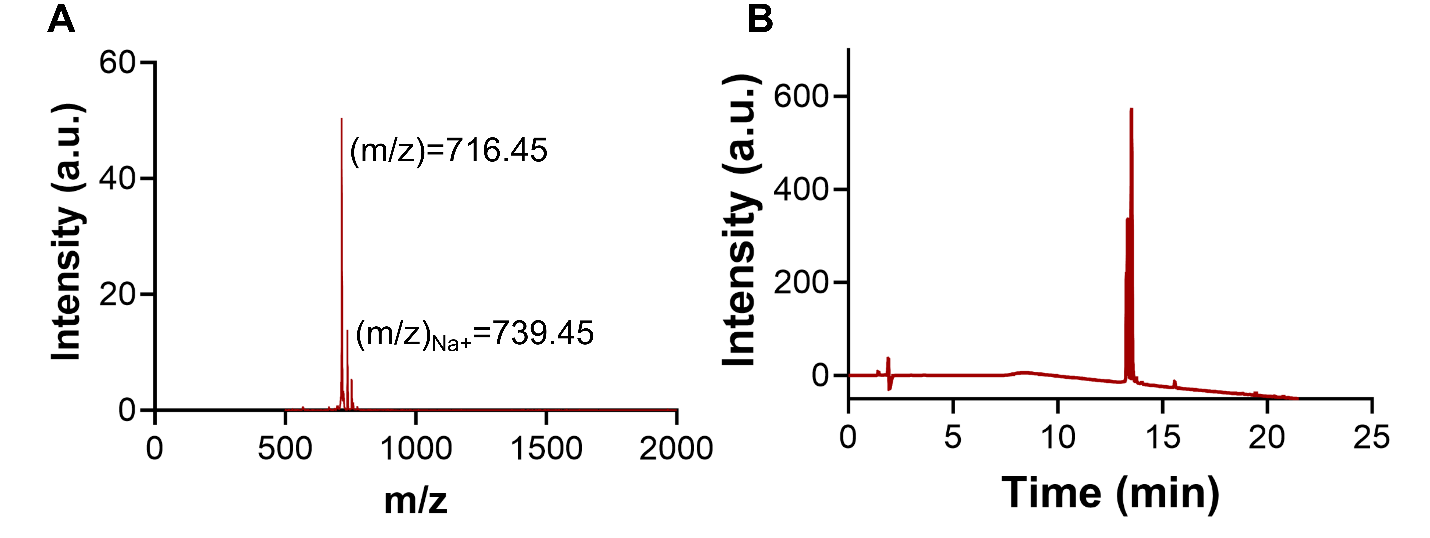


**Figure S3.** Representative (A) MALDI-TOF and (B) HPLC profiles of KFE5(F_G_).


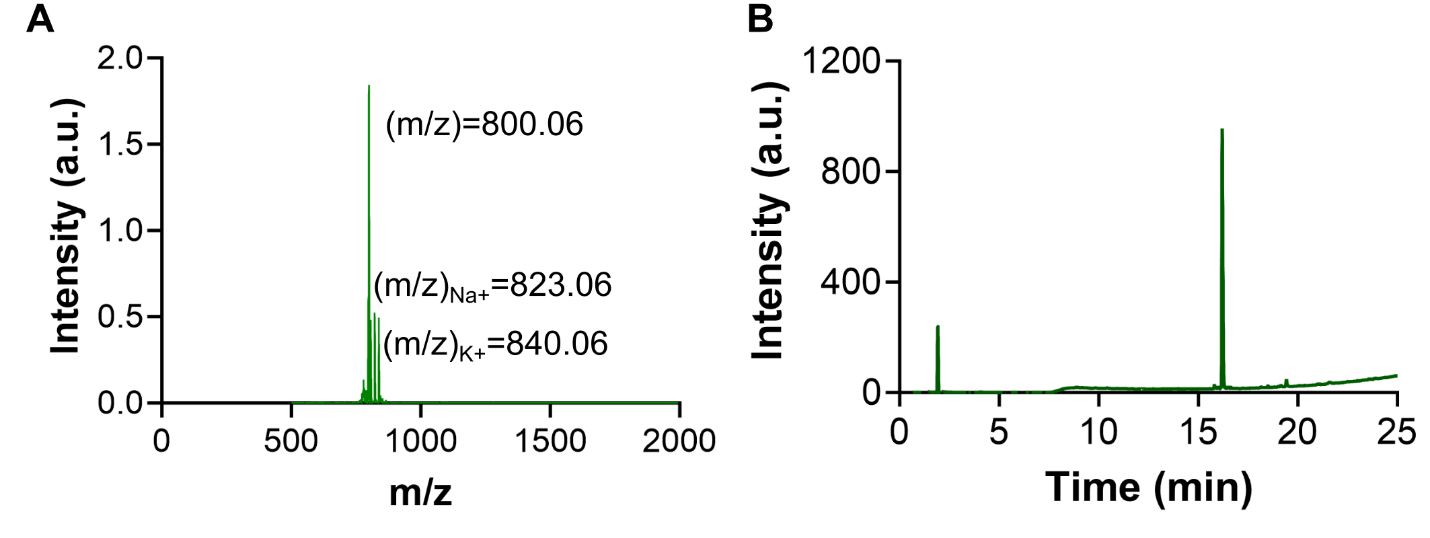


**Figure S4.** Representative (A) MALDI-TOF and (B) HPLC profiles of KFE5(CH_3_).


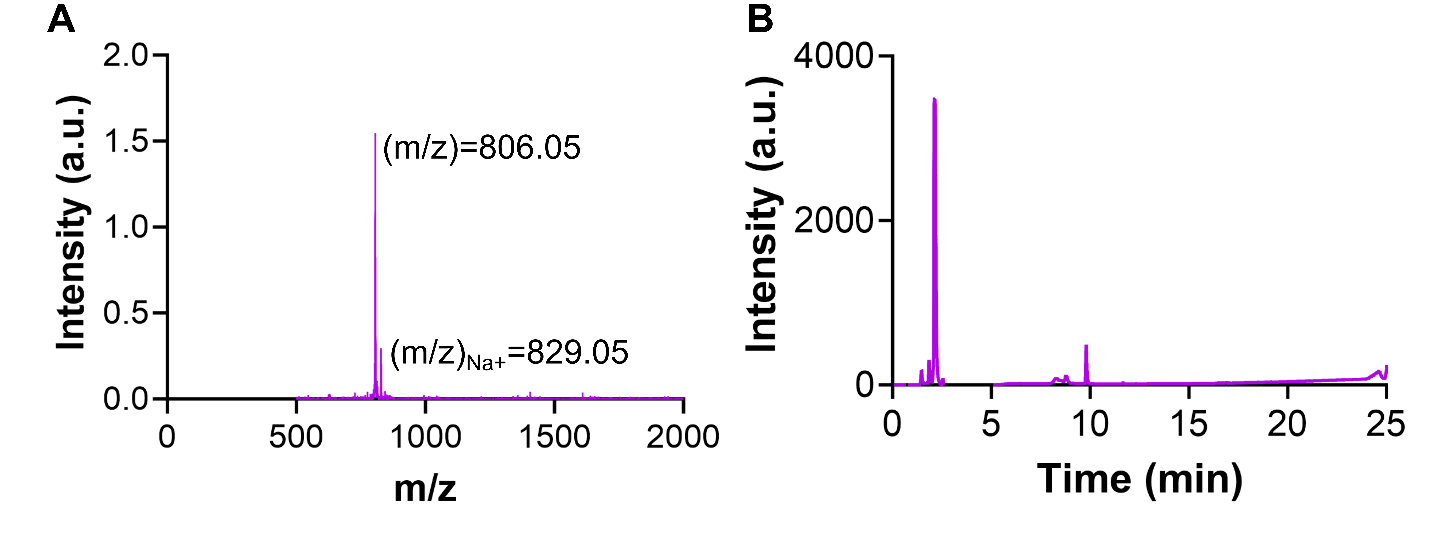


**Figure S5.** Representative (A) MALDI-TOF and (B) HPLC profiles of KFE5(OH).


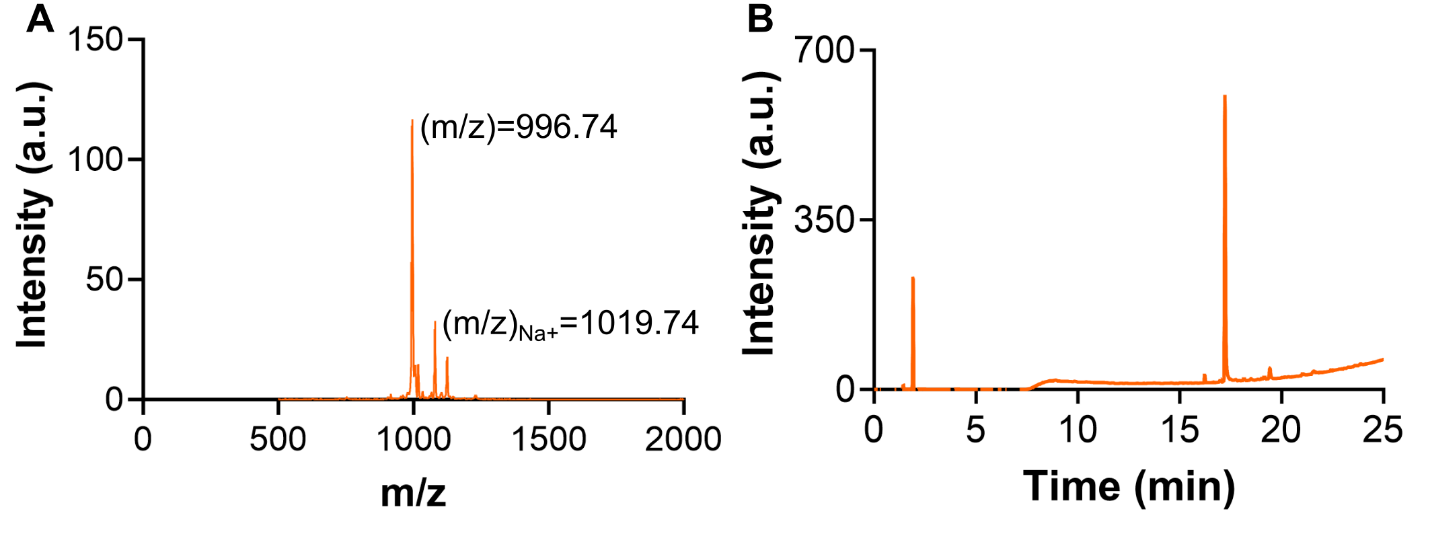


**Figure S6.** Representative (A) MALDI-TOF and (B) HPLC profiles of KFE5(Br).


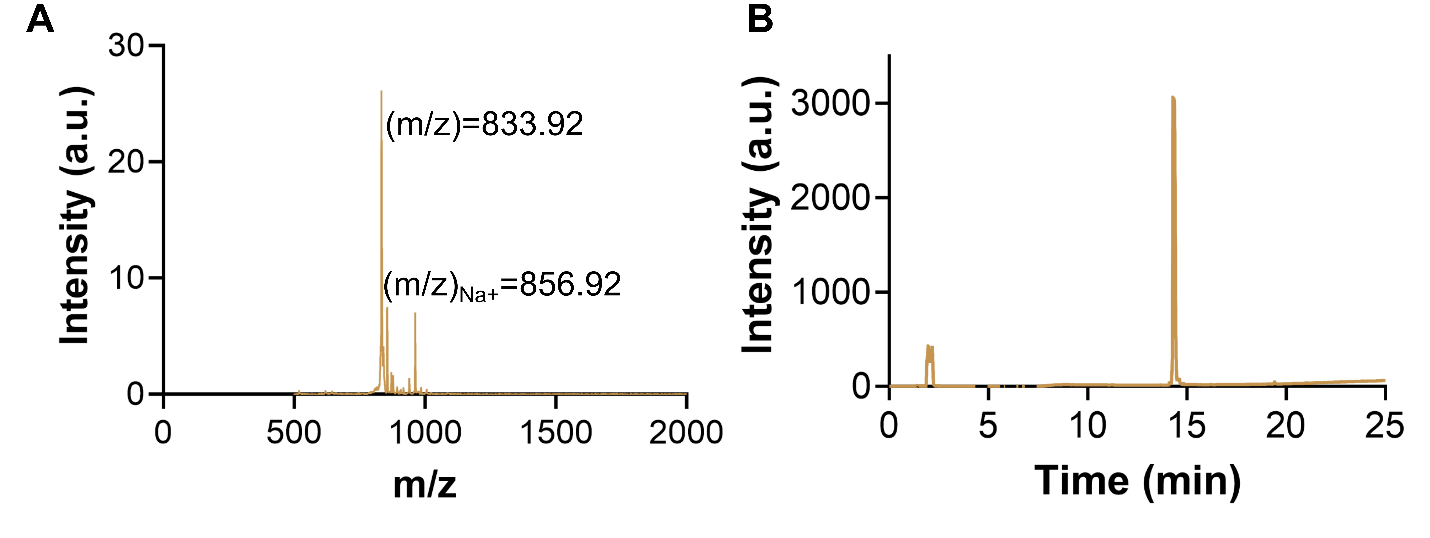


**Figure S7.** Representative (A) MALDI-TOF and (B) HPLC profiles of KFE5(CN).

**
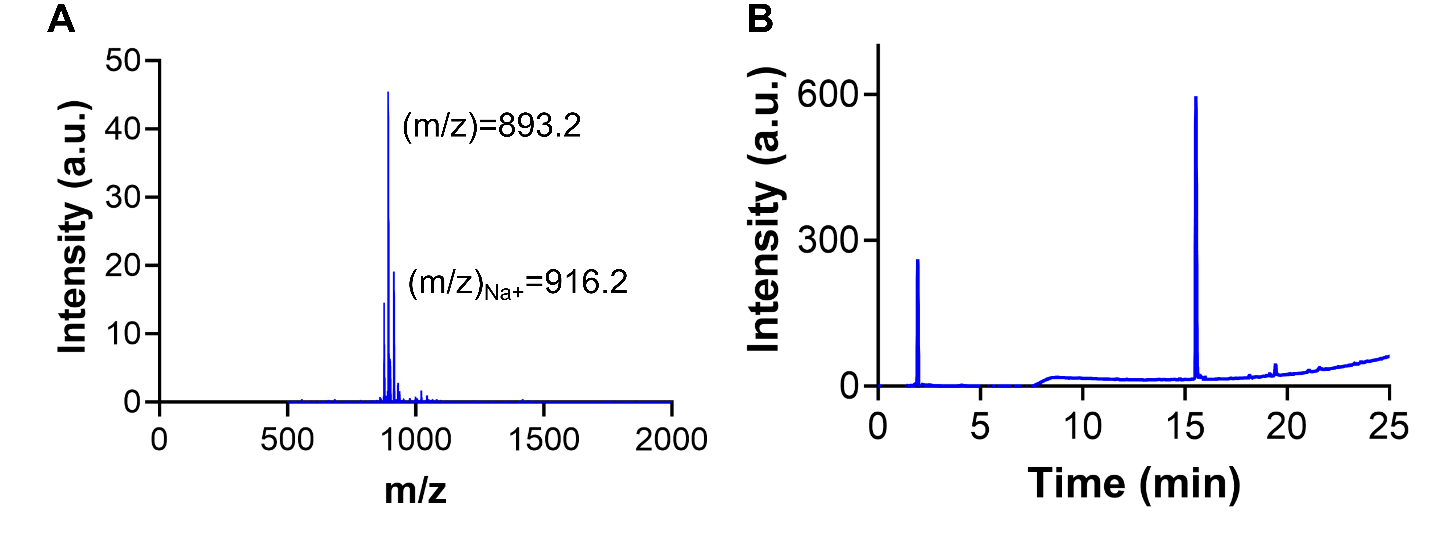
**

**Figure S8.** Representative (A) MALDI-TOF and (B) HPLC profiles of KFE5(NO_2_).


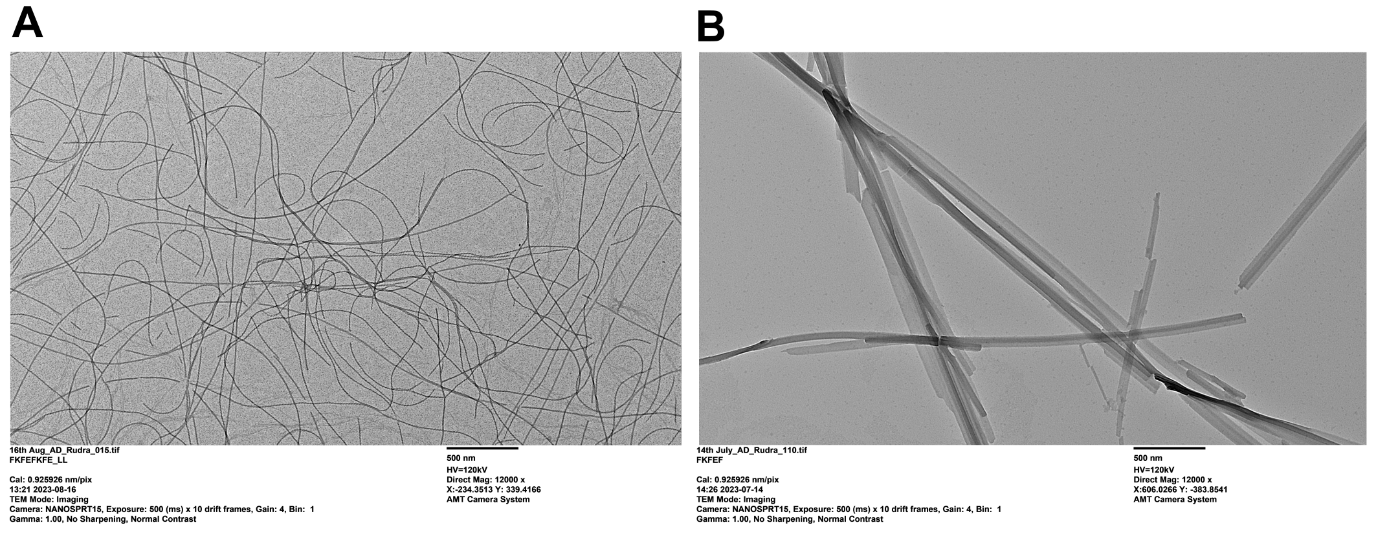


**Figure S9.** Representative TEM images of (A) KFE8 and (B) KFE5 fibers. Scale bar is 500 nm.


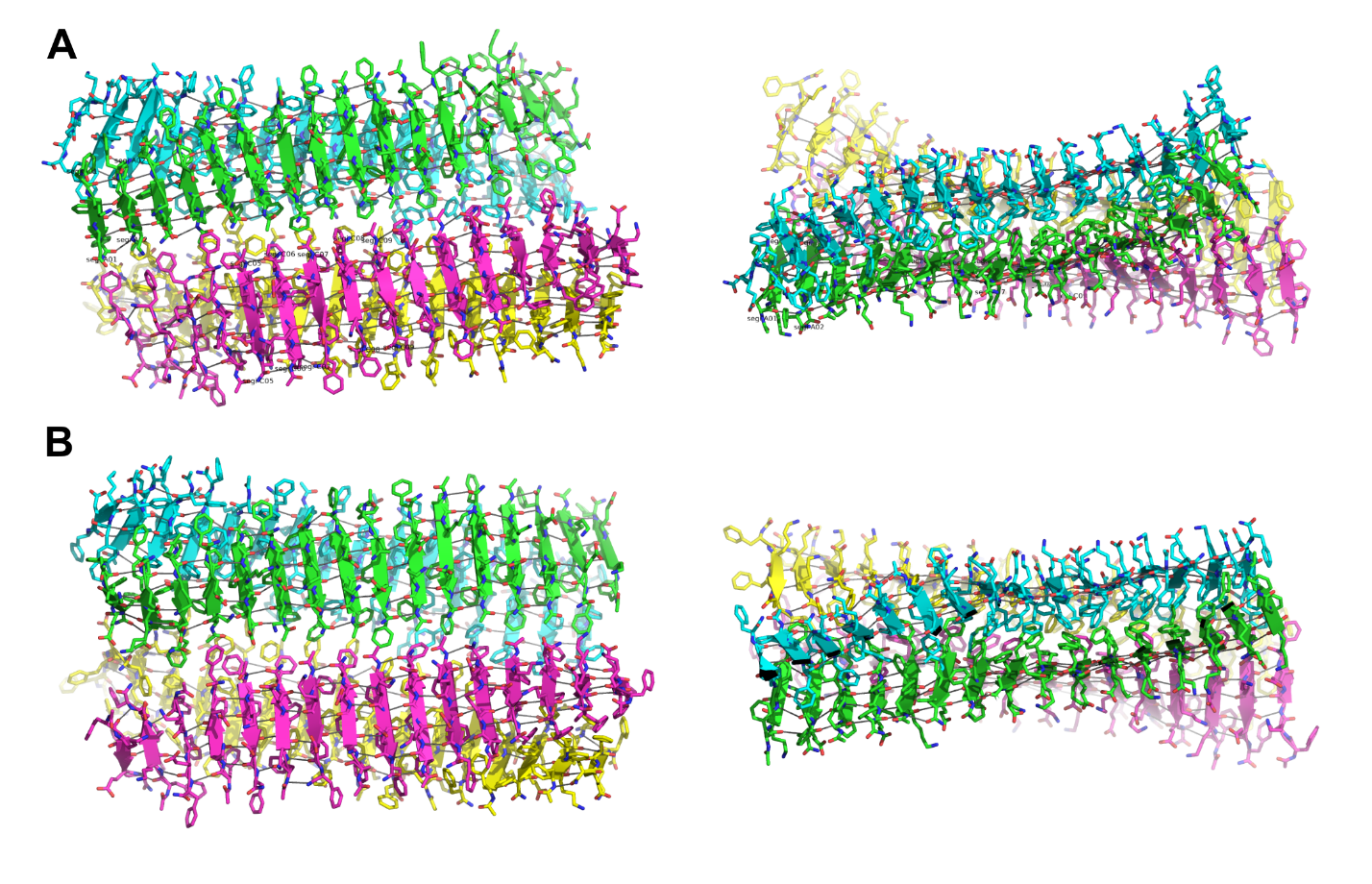


**Figure S10.** A 500 ns MD Simulation of the AlphaFold3 KFE5 model. The model is also based on the H. Wang KFE8 model. It consists of a sandwich of two anti-parallel β-sheet peptide assemblies, each comprising 16 strands that form a fiber. The model features an additional fiber that interacts via the hydrophobic side chains of the fiber edge (Phe:Phe). (A) At frame 14018. (B) At frame 24252.


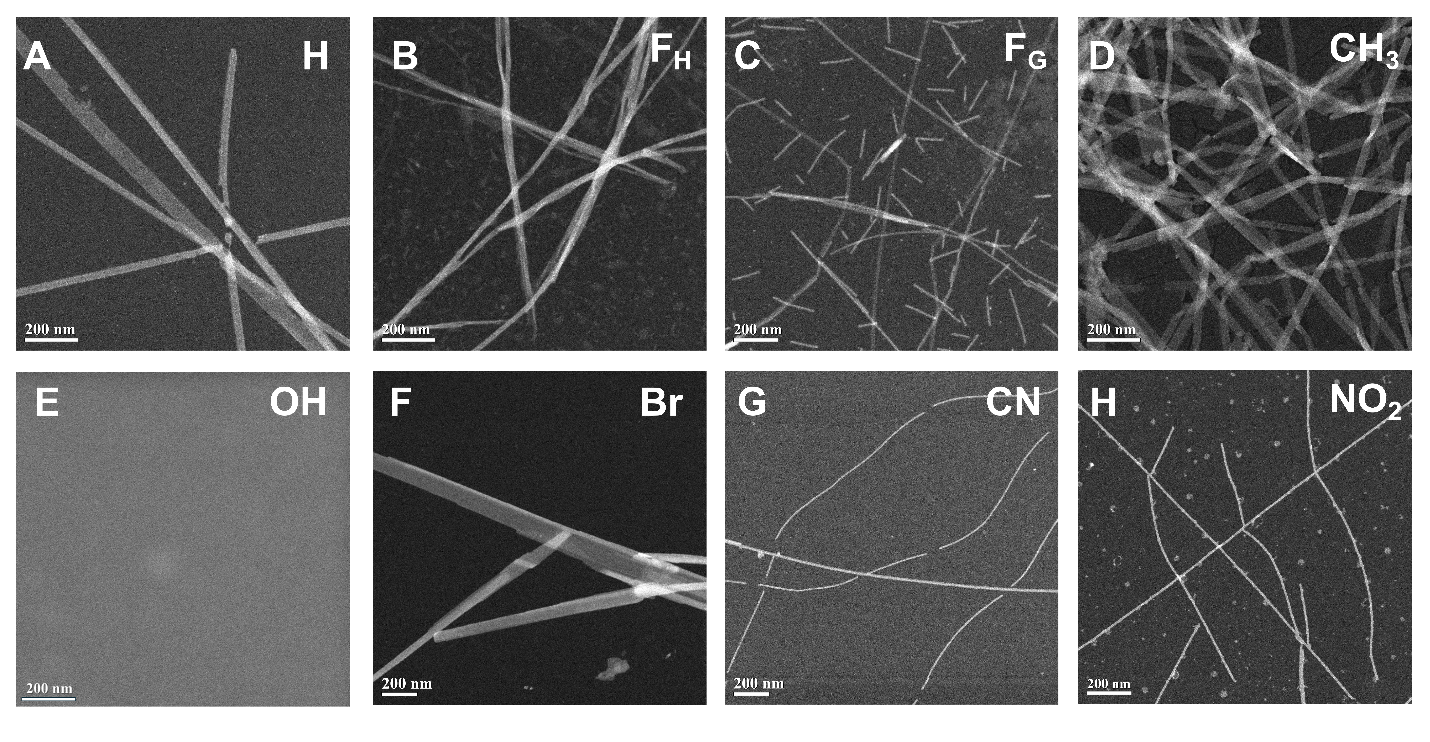


**Figure S11.** Representative TEM images of KFE5 variant peptides. Scale bar is 200 nm.


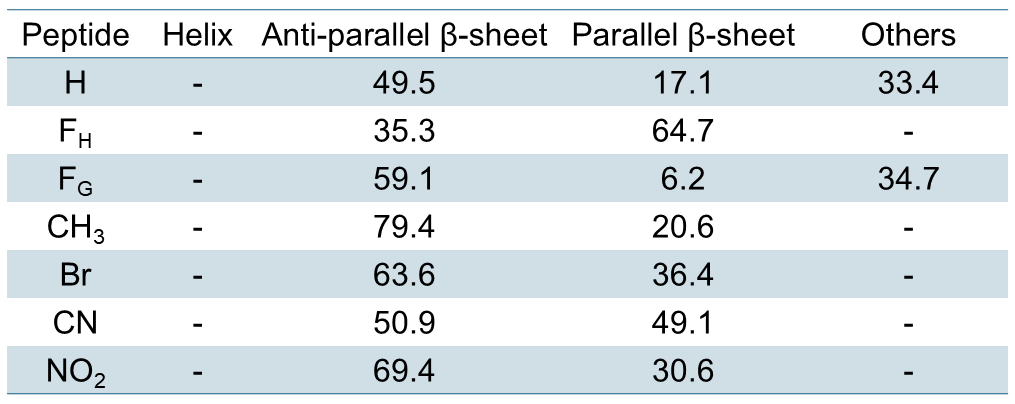
**Table S1:** BeSTSel deconvolution analysis for CD spectra obtained for different KFE5 peptide variants.


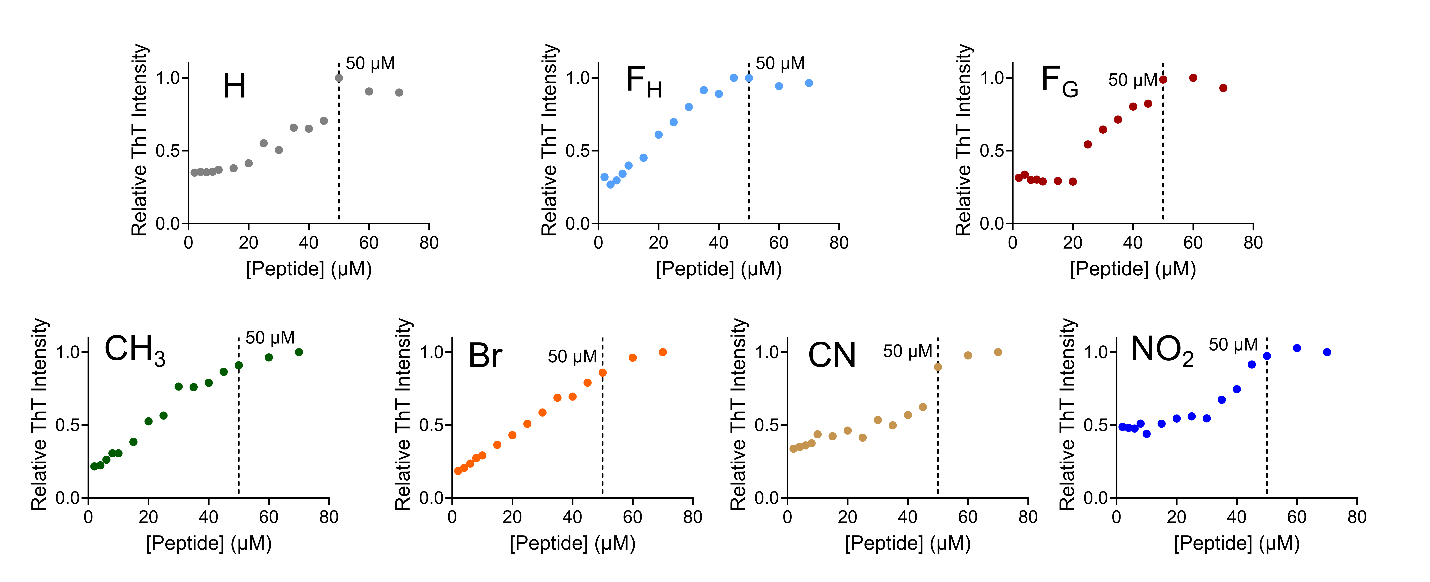


**Figure S12.** Concentration-dependent relative ThT fluorescence for all peptides at [ThT]= 10 μM.


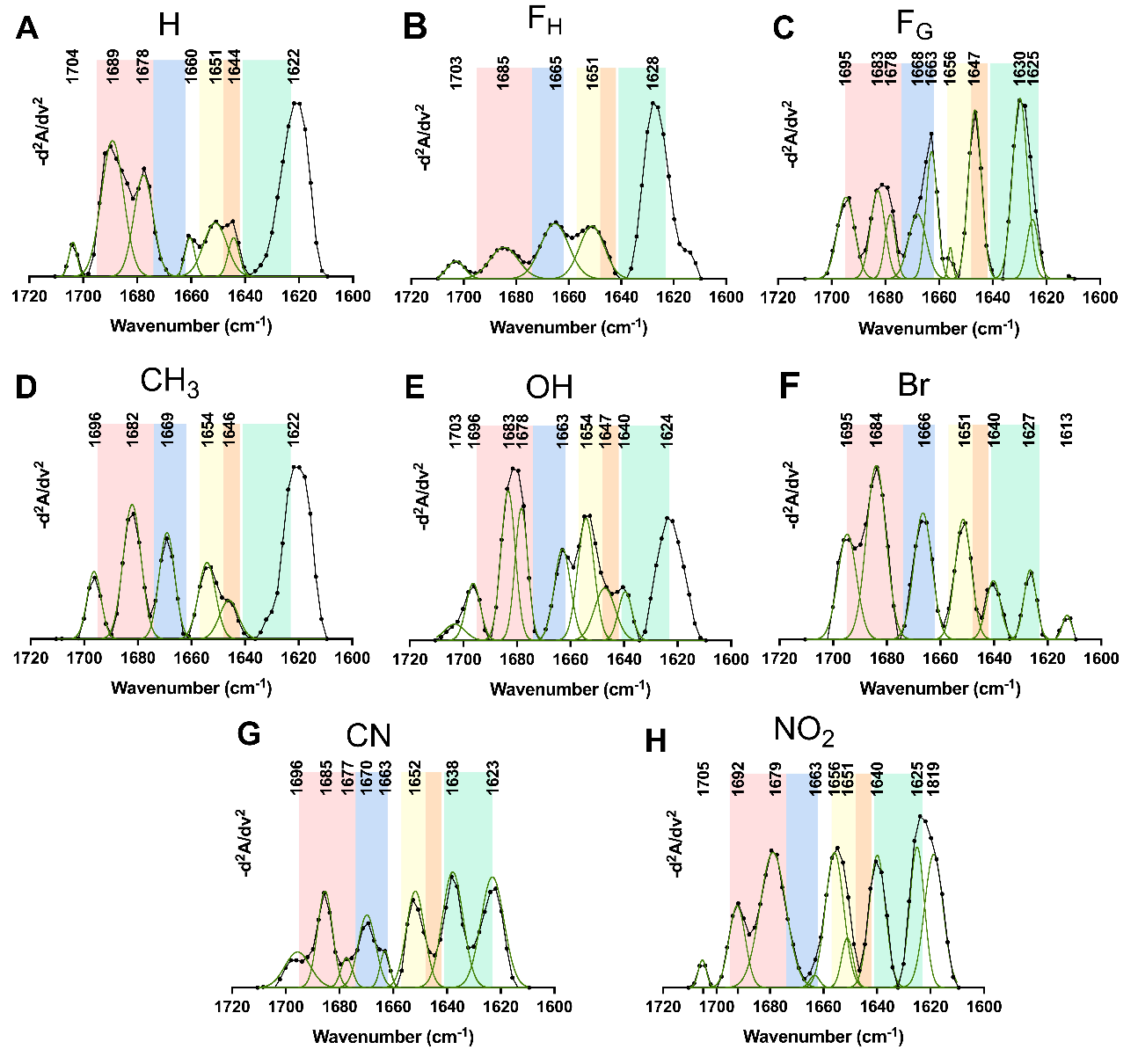


**Figure S13.** Second-derivative FT-IR spectra of KFE5 variants (A)-(H) in ultra-pure biological grade water.

**
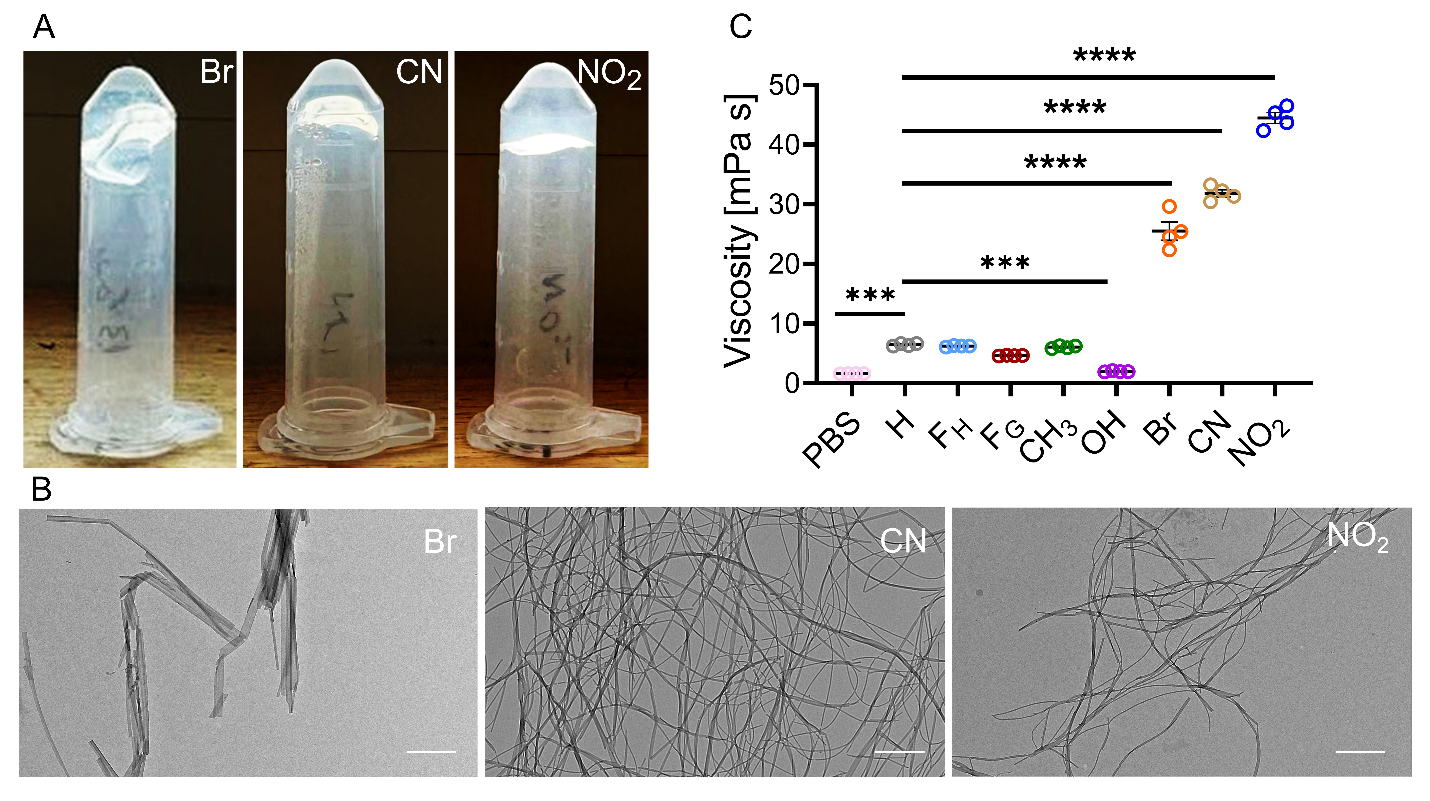
Figure S14.** (A) Representative images of gel formation by KFE5(Br) (2.5 mM), KFE5(CN) (5 mM), and KFE5(NO_2_) (5 mM) peptides. (B) TEM images depicting network-like fibrillar structures for KFE5(Br), KFE5(CN), and KFE5(NO_2_) peptides. Scale: 500 nm. (C) Viscosity measurements of peptide solutions at a shear rate of 100 s^-1^. ***p < 0.001, ****p < 0.0001 as determined by a one-way ANOVA (n=4).


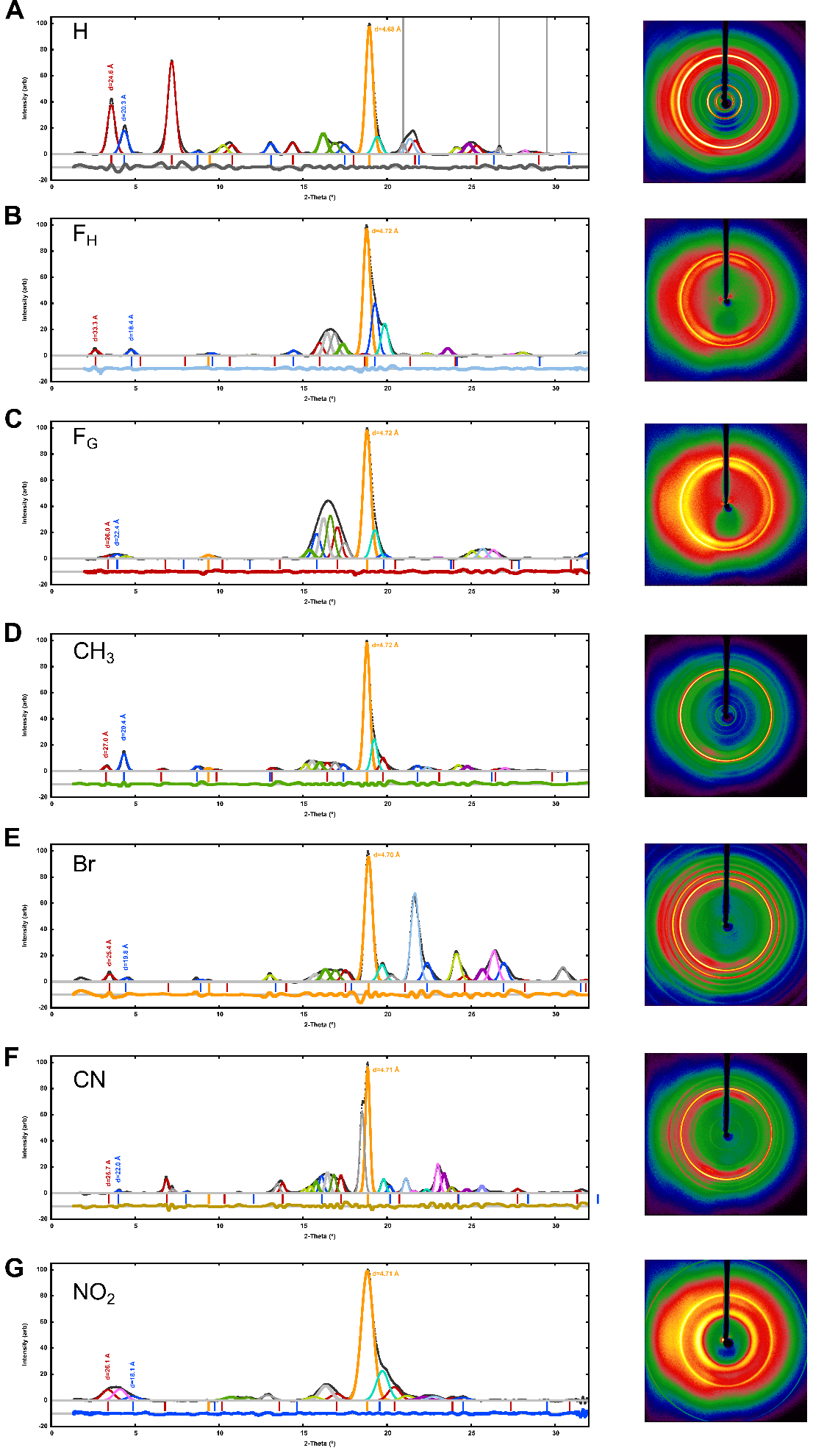


**Figure S15.** (A) Fitting of the KFE5 XRD (powder diffraction) data along with its image (scan width 36°). Major peaks are (▬) β-sheet (d=4.693(3) Å), and (▬) helical repeat (d=24.751(5) Å) with 4 higher-order peaks at d=12.4, 8.25, 6.19, and 4.95 Å. Another (▬) major peak at d=20.38(2) Å has a possible (▬) (n=2) repeat at d=10.19 Å, (n=3) at d=6.79 Å, and n=4 at d=5.09 Å. Additional peaks occur at (▬) d=8.64(1), (▬) d=5.480(4), (▬) d=5.259(8), (▬) d=3.698(3), (▬) d=3.580(3), (▬) d=3.258(7). The (-) narrow peaks appear to be SiO_2_ from the capillary mounting glue. The (▬) residual is shown below. The scattering background was removed using an 18-parameter polynomial background fit in d1Dplot. The KFE5 sample was dissolved in methanol, pipetted into a MiTeGen capillary, and then left to dry prior to data collection. The KFE5 diffraction image, from a 360° φ-scan (right).

(B) The KFE5(F_H_) XRD powder diffraction peak fitting, along with the diffraction image in the right panel.

(C) The KFE5(F_G_) XRD powder diffraction peak fitting, along with the diffraction image in the right panel.

(D) The KFE5(CH_3_) XRD powder diffraction peak fitting. along with the diffraction image in the right panel.

(E) The KFE5(Br) sample exhibits major peaks in common with KFE5. The β-sheet peak is at 2θ~19° (d=4.69 Å), the first helical twist peaks (d=24.7 Å) is seen at 2θ~ 3.5°, 7.13°, (10.7°), (14.4°), and 21.5°. The second helical twist (d=20.4 Å) is observed at 2θ~ 4.36° (8.7°), 13.0°, 17.5°. The large peak from 14.0° to 18.0° is due to the capillary holder and is much larger in the KFE5(Br) data due to a smaller fraction of sample powder. The (▬) residual is shown below. The KFE5(Br) sample was dissolved in water, pipetted into a MiTeGen capillary, and then left to dry prior to data collection. (right) The KFE5(Br) diffraction image, from a 360° φ-scan.

(F) The KFE5(CN) XRD powder diffraction peak fitting. The main (▬) d=4.75(1) Å β-sheet peak is dominant. The filament structure has changed and is missing the strong (▬) d=24 Å feature. Two new peaks at (▬) d~13.41(1) Å and (▬) d~6.619(2) Å have appeared. Only two (▬) d~25.46(1) higher order peaks at n=2, d~12.7 Å and n=4, d~6.36 are possibly observed. The other helical (▬) d~19.36(3) Å reflections are possibly observed at n=4, d~ 4.84 Å, and n=5, d~ 3.87 Å. (right) The KFE5(CN) diffraction image, from a 360° φ-scan.

(G) The KFE5(NO_2_) XRD powder diffraction peak fitting, along with the diffraction image on the right panel.

**
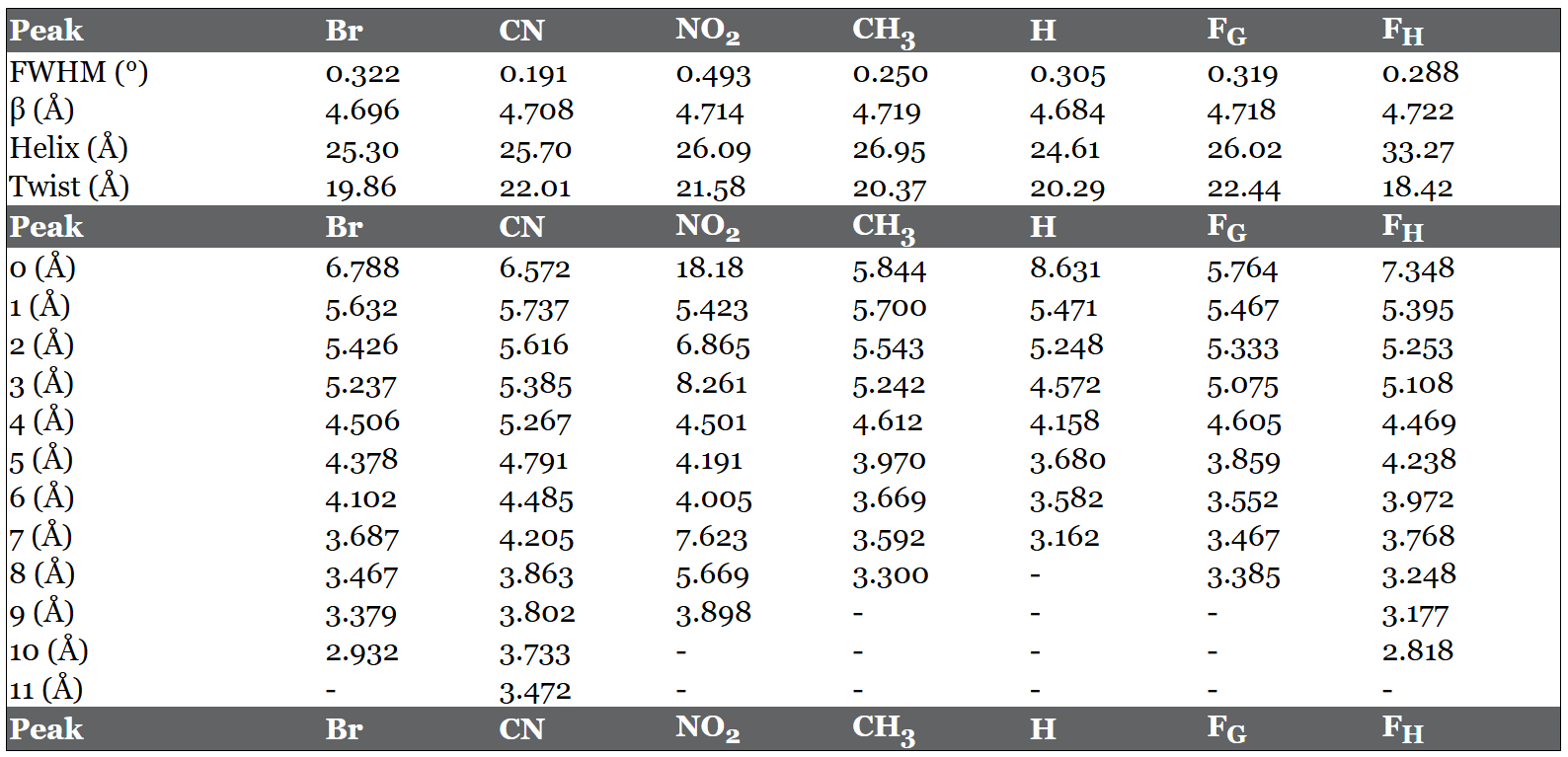
Table S2:** WAXS peaks for 5-mer peptides.


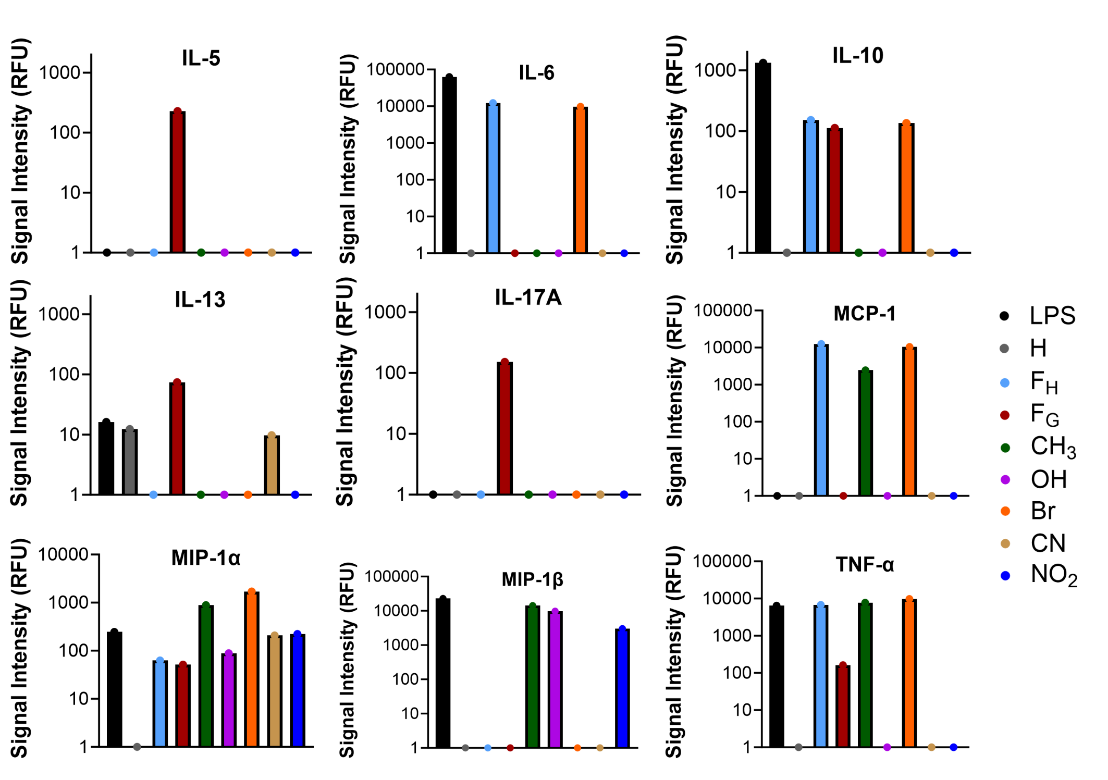
**Figure S16.** Cytokines and chemokines production in mouse bone-marrow dendritic cells (DCs) treated with peptide nanofibers (10 μM) for 24 h.


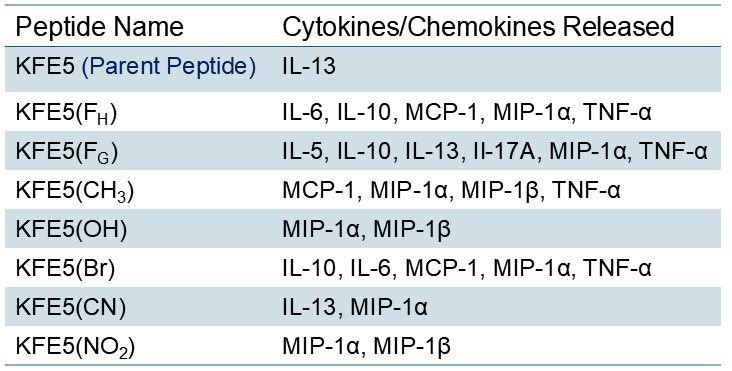
**Table S3:** Comprehensive list of chemokines and cytokines released upon KFE5 (X) treatments.


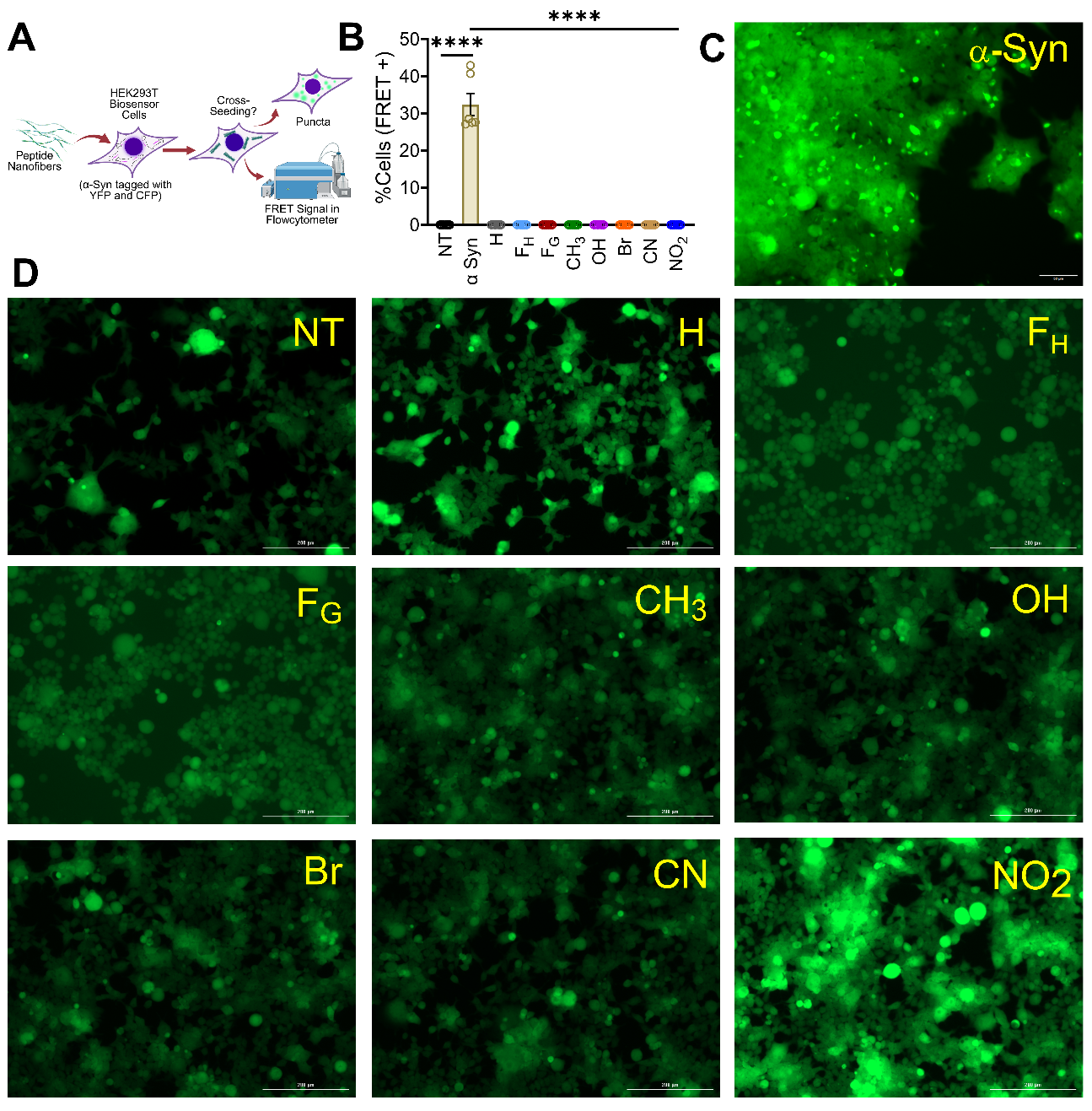


**Figure S17.** (A) Schematic representation of FRET assay in HEK293T biosensor cells in the presence of 5-mer peptide nanofibers. (B) % FRET positive cells as determined by the assay at 50 nM nanofiber concentration compared to α-synuclein PFFs (50 nM). *p < 0.05, **p < 0.01, ***p < 0.001, ****p < 0.0001 as determined by a one-way ANOVA (n=6). (C) Representative microscopy images of 50 nM α-Syn and (D) 5-mer PNFs added to biosensor cells. The green punctae represent FRET due to aggregation caused by α-syn PFFs to monomeric α-syn. Scale bar is 200 nm.

**Table S4:** Sequences and abbreviations for OVA-conjugated peptides used in this study.


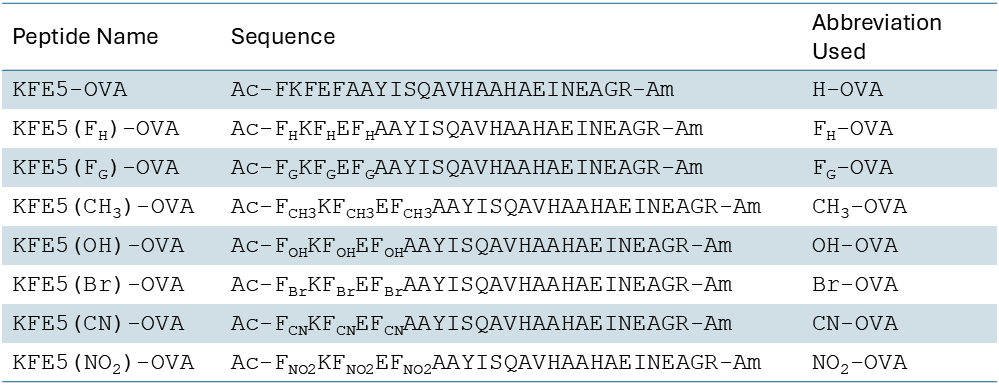


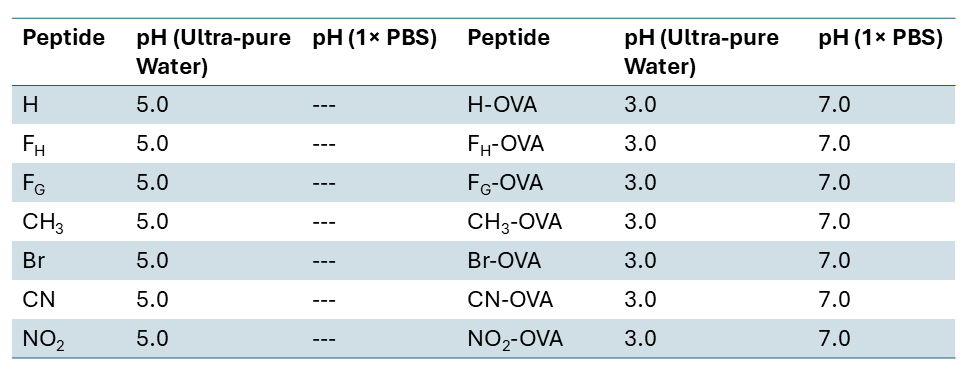
**Table S5:** pH measurements of peptide solutions used in the *in vitro* and *in vivo* studies.


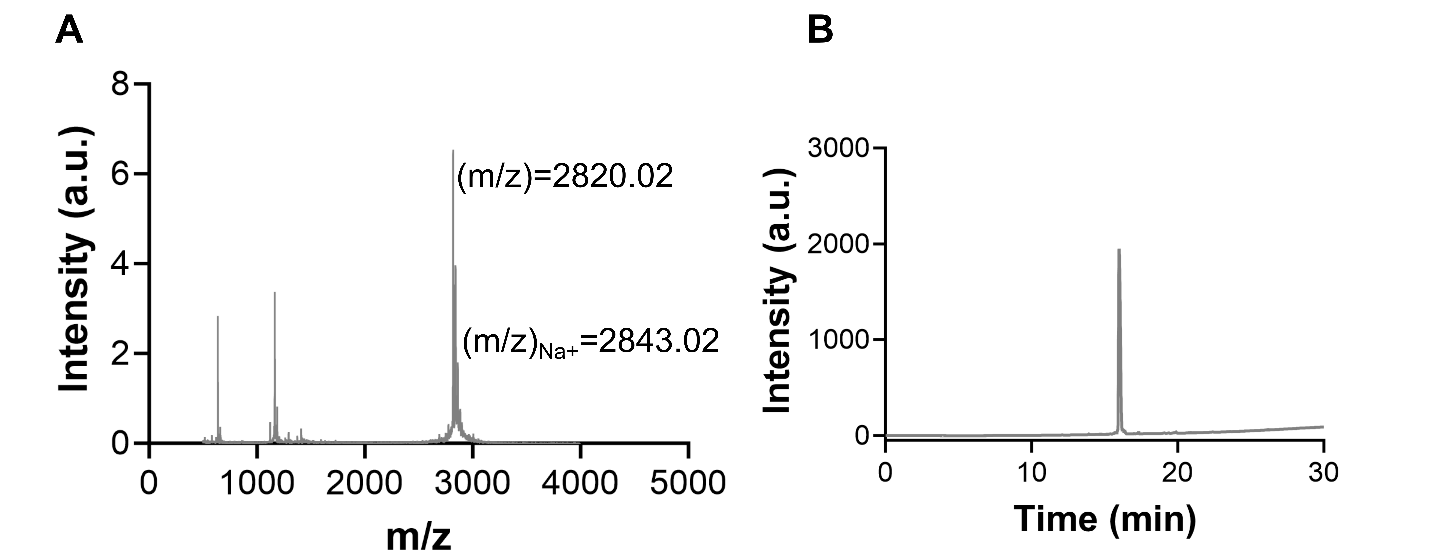


**Figure S18.** Representative (A) MALDI-TOF and (B) HPLC profiles of KFE5-OVA.


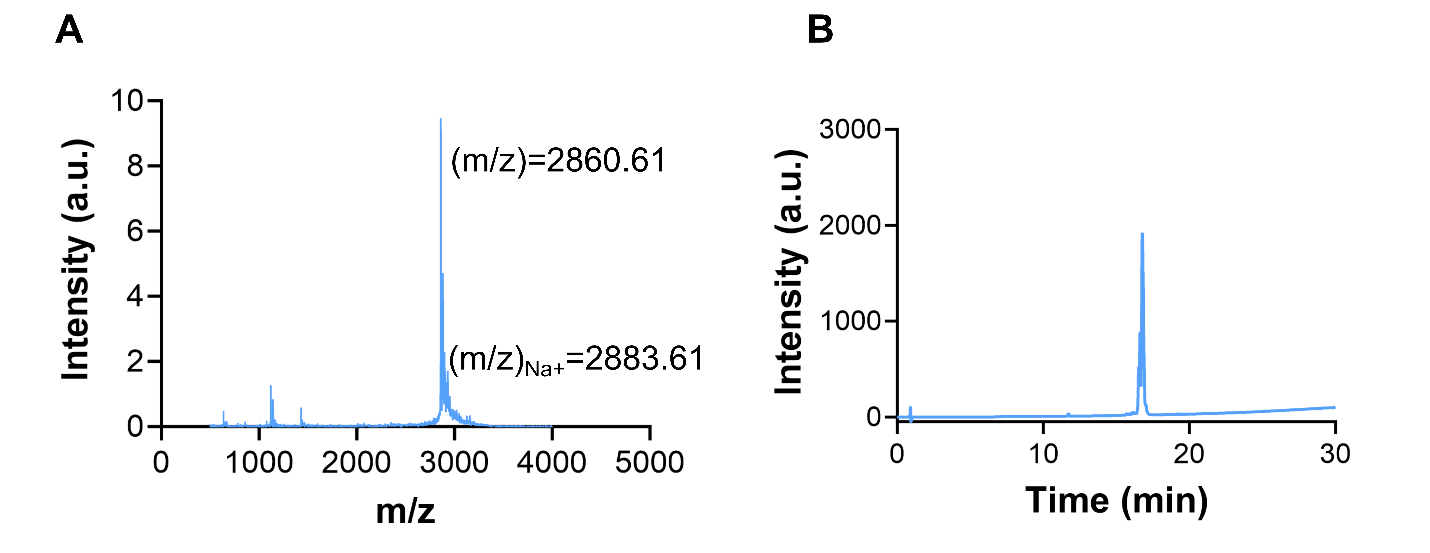


**Figure S19.** Representative (A) MALDI-TOF and (B) HPLC profiles of KFE5(F_H_)-OVA.


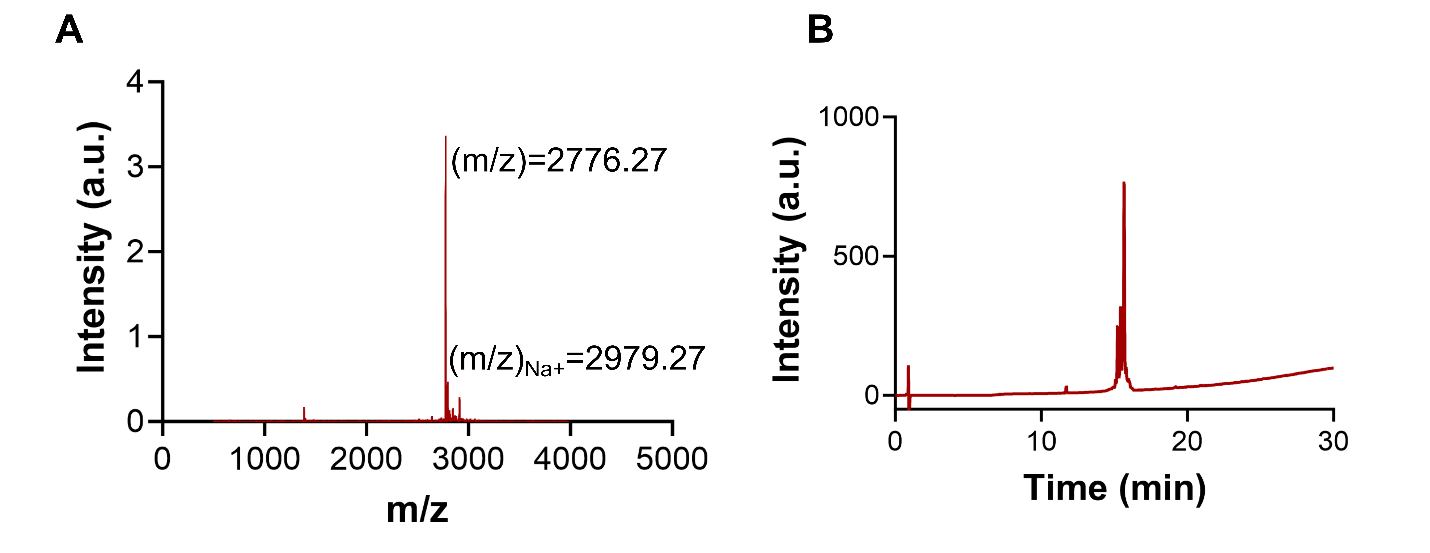


**Figure S20.** Representative (A) MALDI-TOF and (B) HPLC profiles of KFE5(F_G_)-OVA.


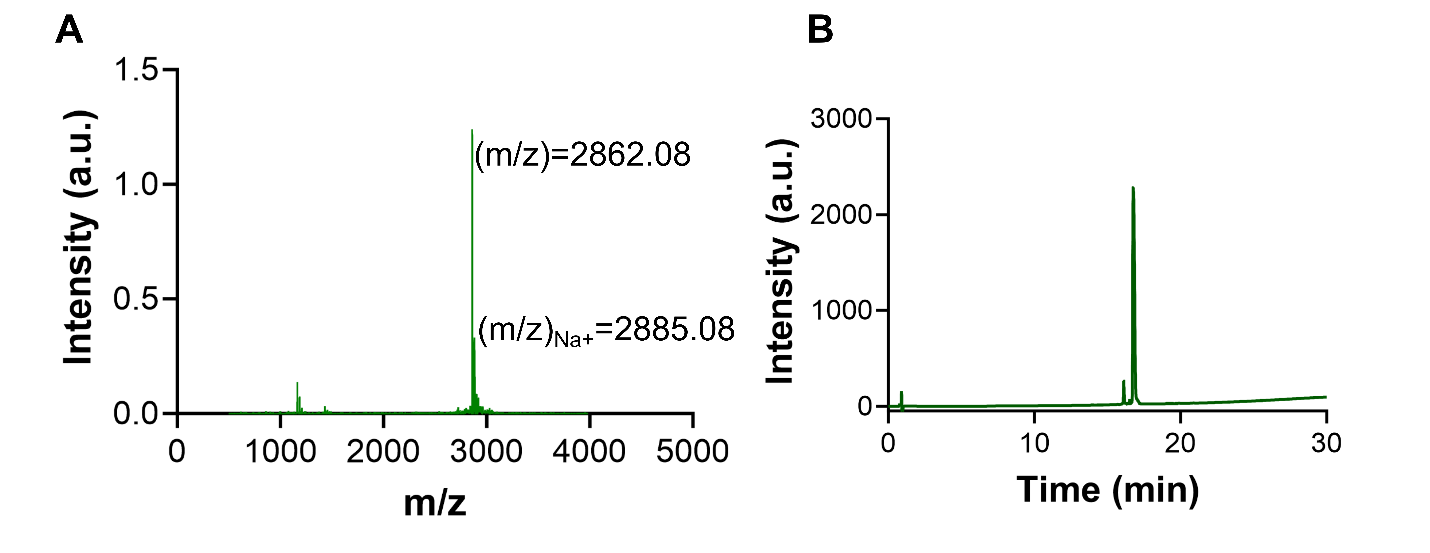


**Figure S21.** Representative (A) MALDI-TOF and (B) HPLC profiles of KFE5(CH_3_)-OVA.


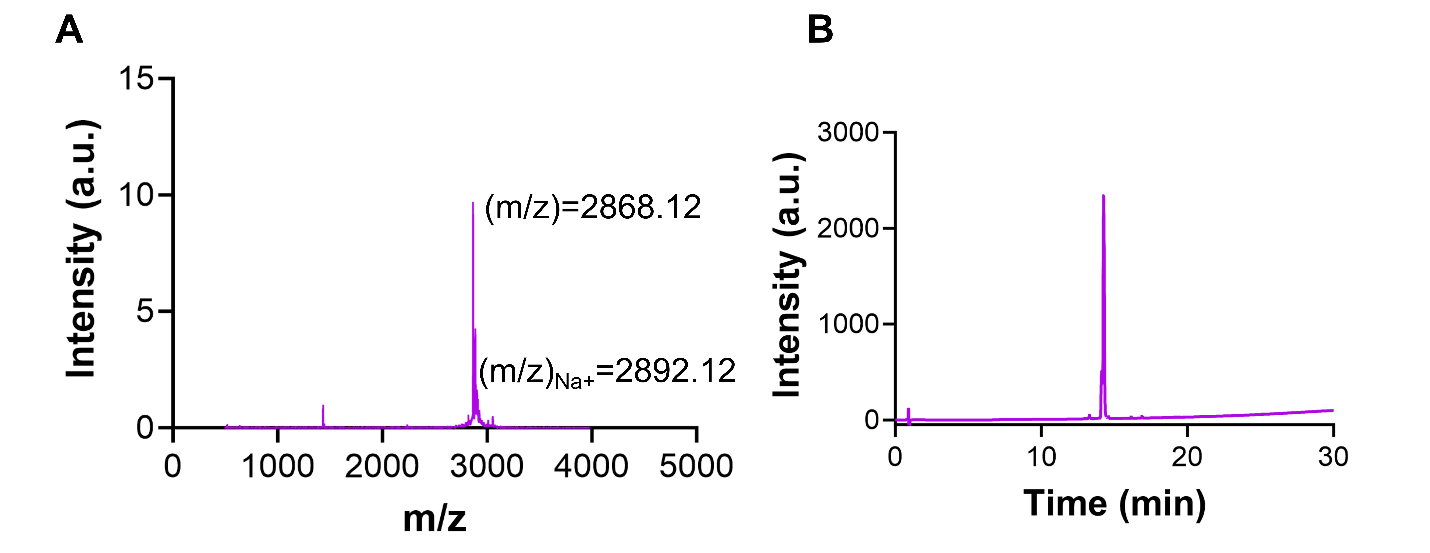


**Figure S22.** Representative (A) MALDI-TOF and (B) HPLC profiles of KFE5(OH)-OVA.


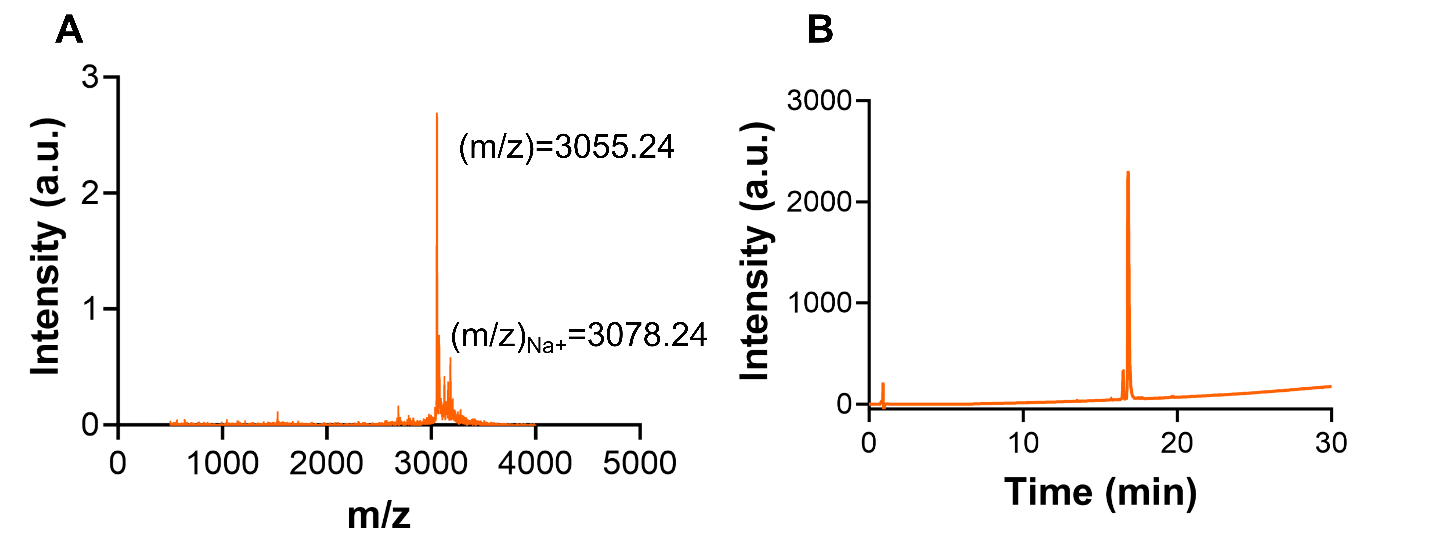


**Figure S23.** Representative (A) MALDI-TOF and (B) HPLC profiles of KFE(Br)-OVA.


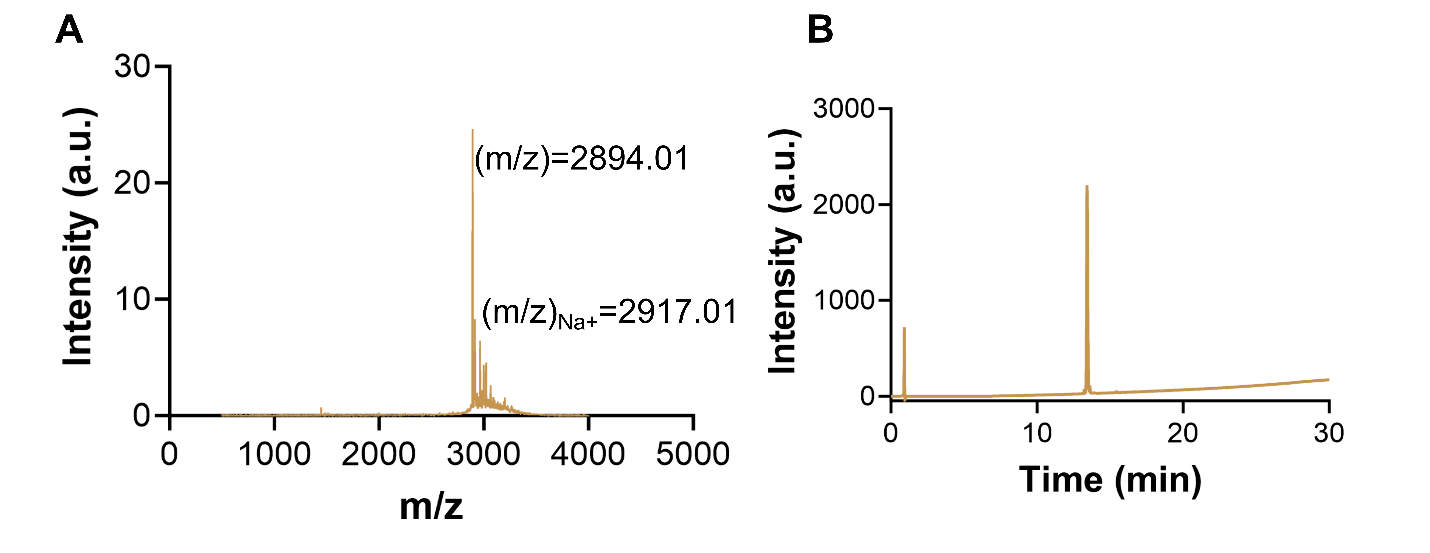


**Figure S24.** Representative (A) MALDI-TOF and (B) HPLC profiles of KFE5(CN)-OVA.


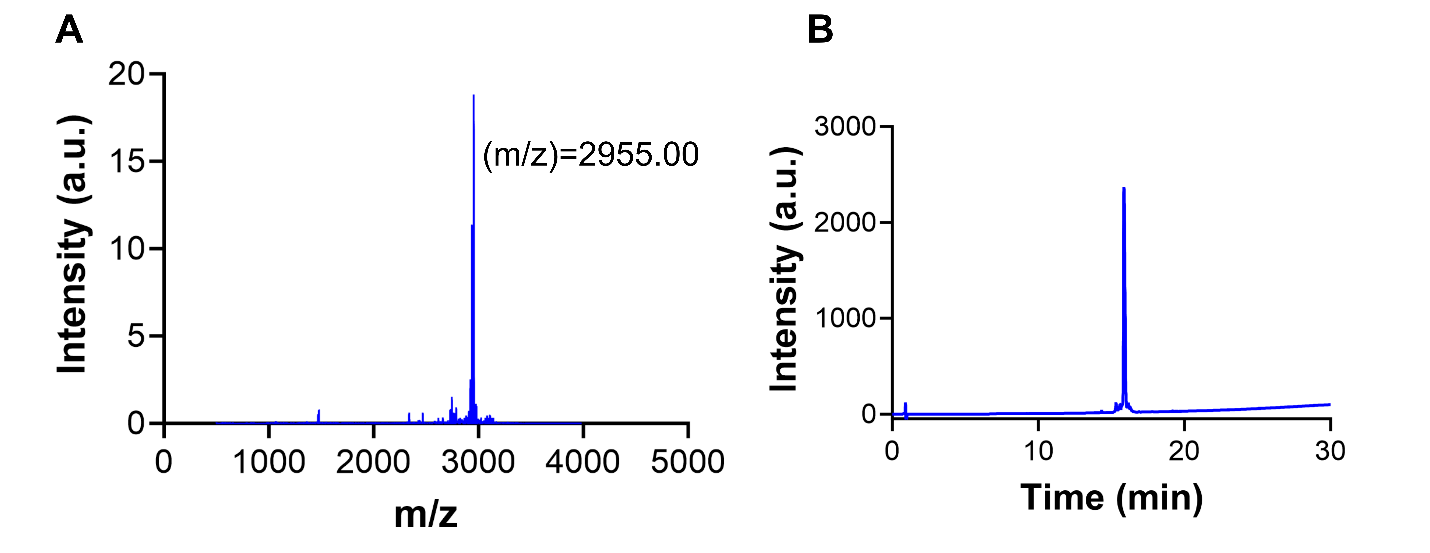


**Figure S25.** Representative (A) MALDI-TOF and (B) HPLC profiles of KFE5(NO_2_)-OVA.

**
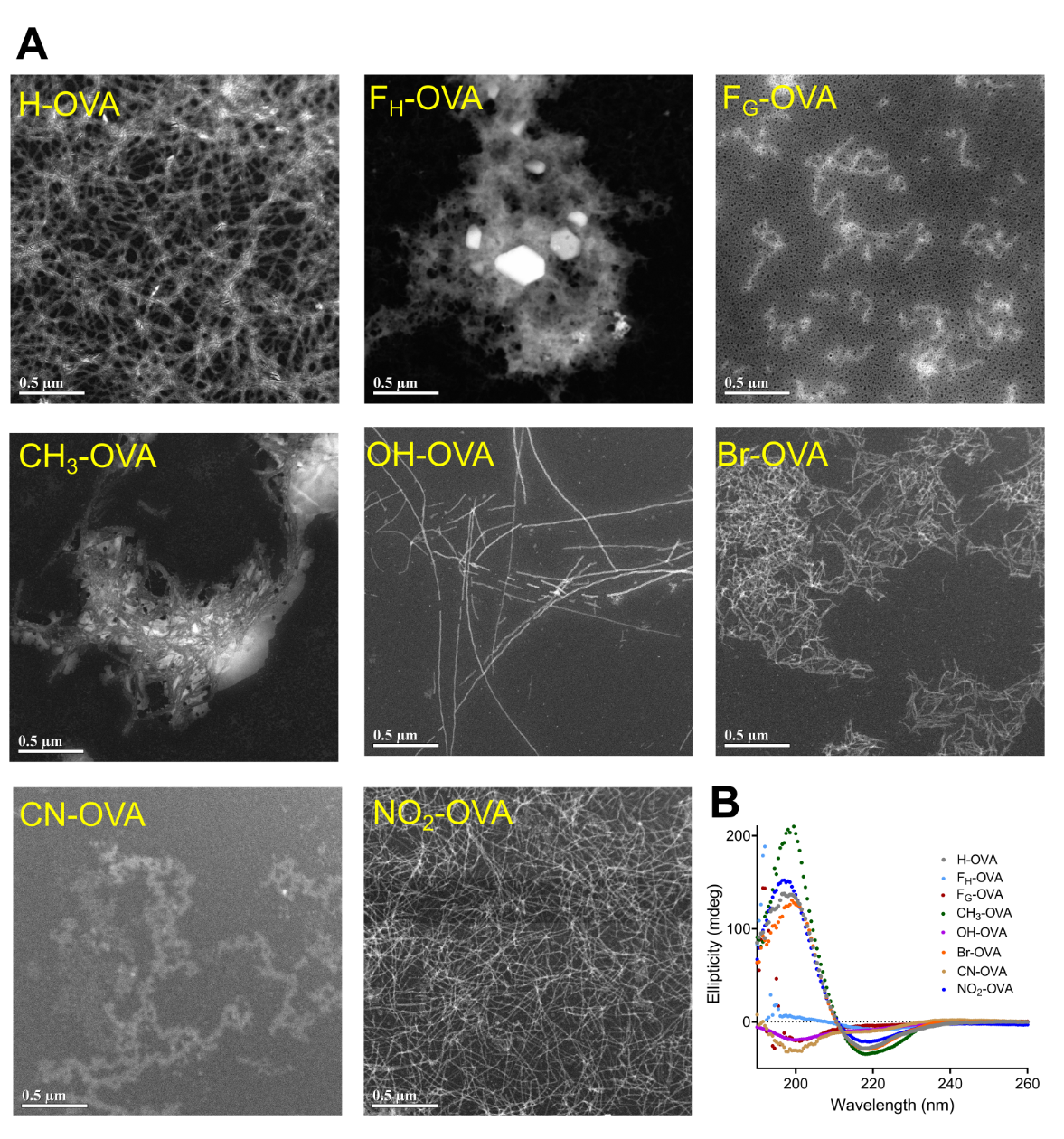
**

**Figure S26.** (A) Representative TEM images of self-assembled OVA-conjugated pentapeptides, as marked in the figure. (B) CD profiles of the OVA-conjugated peptide fibrils.


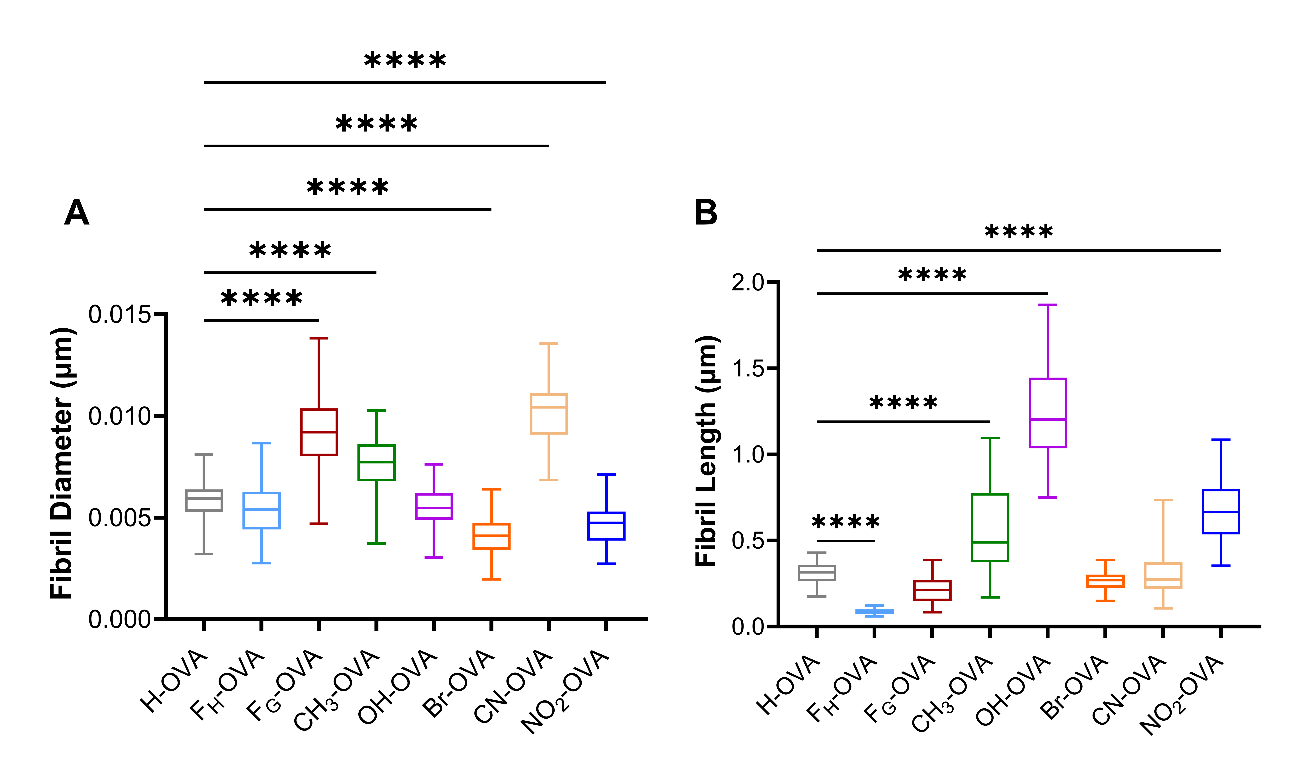


**Figure S27.** Diameter and length of OVA-KFE5 variants (n=75). ****p < 0.0001, determined by ANOVA.


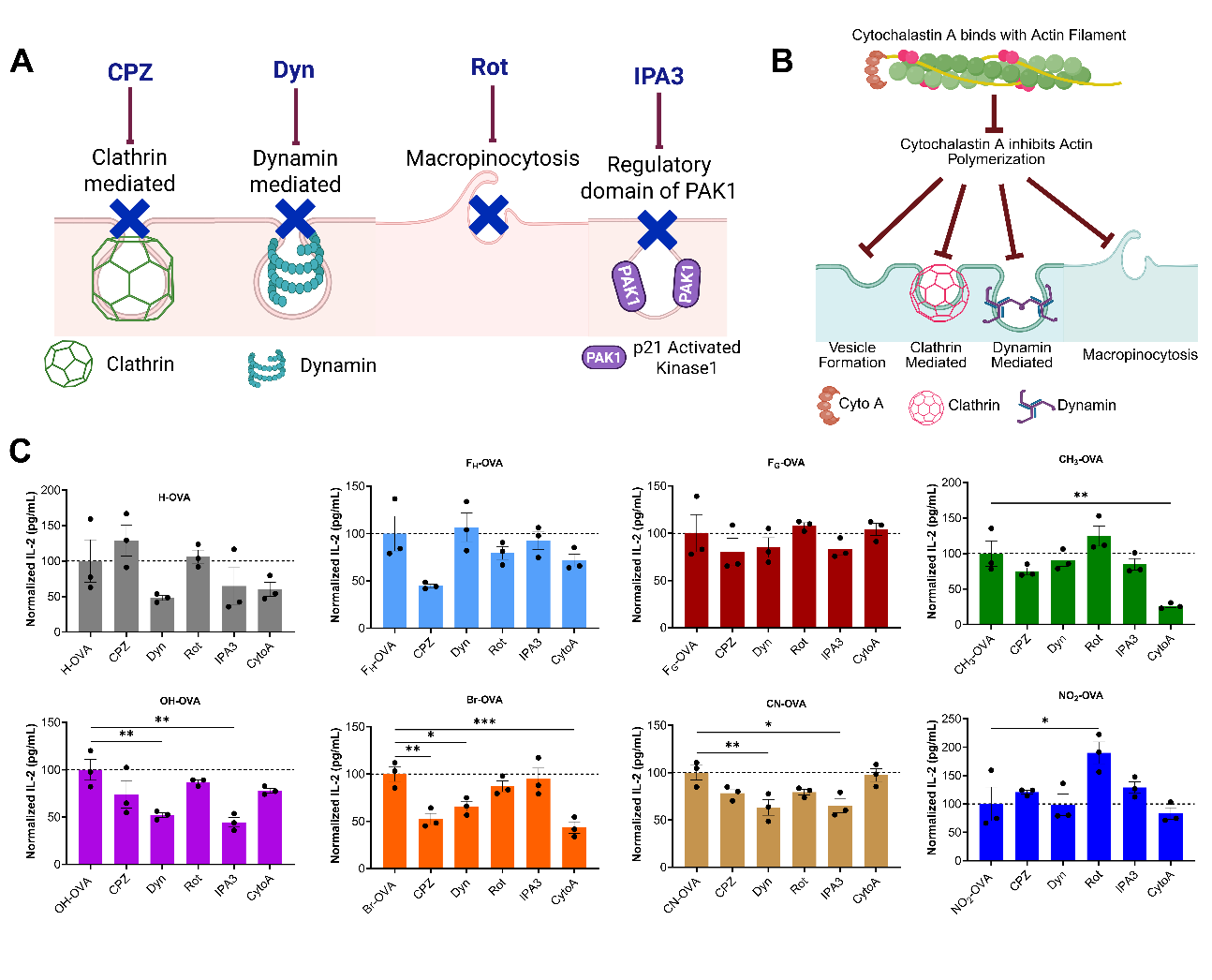
**Figure S28.** Schematic of the mechanisms of action of (A) CPZ, Dyn, Rot, IPA3, and (B) CytoA. (C) Effect of endocytic inhibitors on the uptake of OVA-conjugated KFE5 variants. *p < 0.05, **p < 0.01, ***p < 0.001, determined by ANOVA.


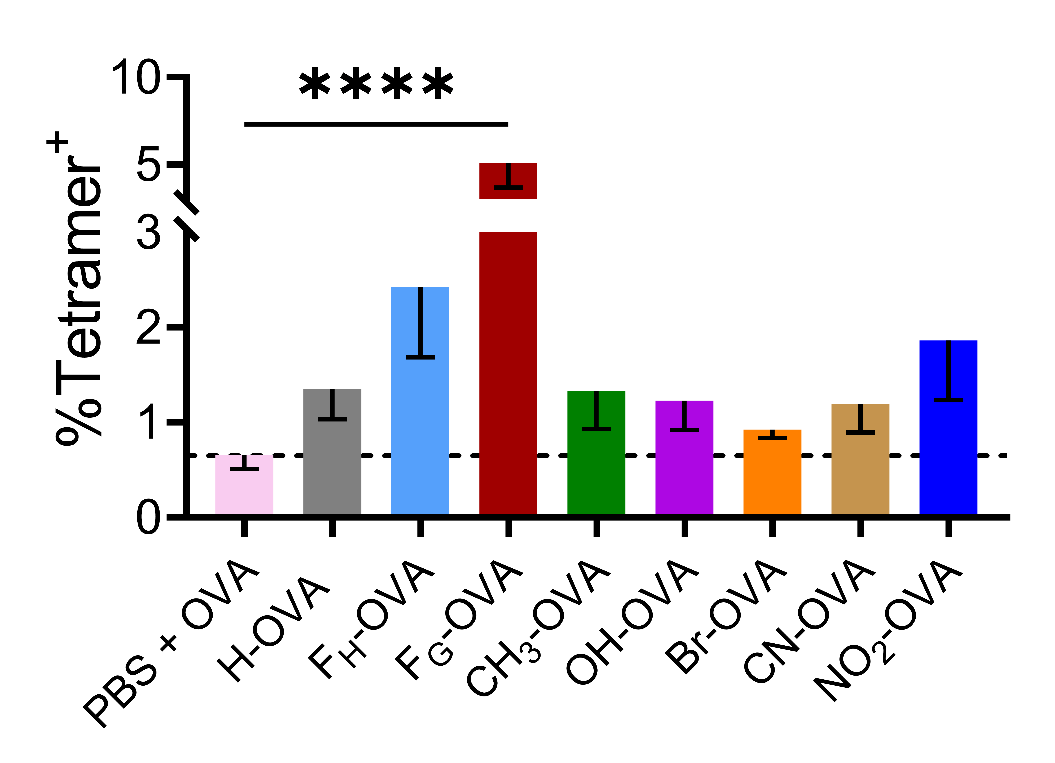


**Figure S29.** The frequency of OVA-specific CD4^+^ T cells is represented as a percentage of total CD4^+^ T cells in the spleens of vaccinated mice. Data is represented as Mean±SEM. ****p < 0.0001 (n=6), as determined by a one-way ANOVA.


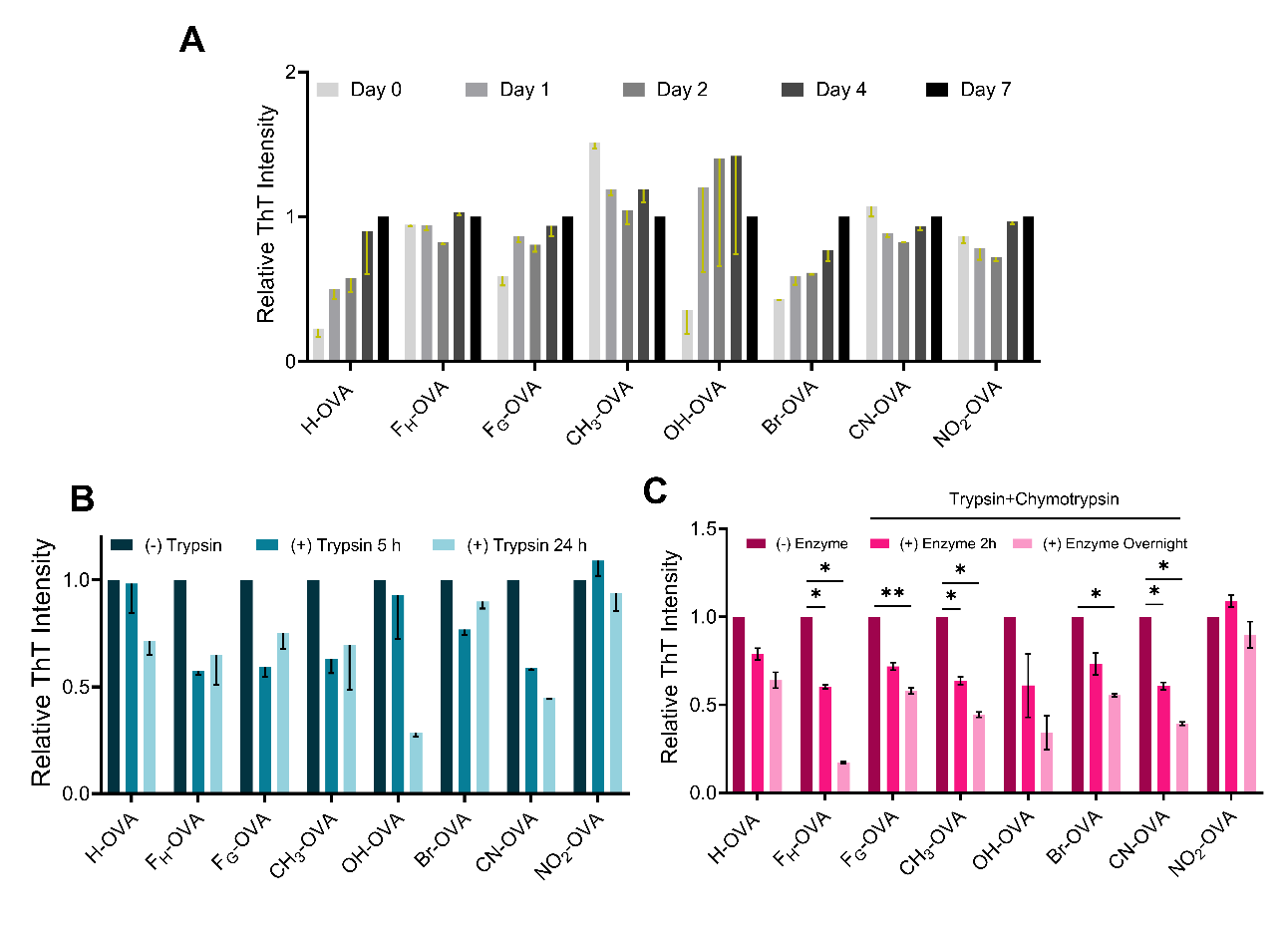


**Figure S30.** (A) Relative ThT intensity of OVA-conjugated KFE5 variants monitored over 7 days. Proteolytic stability of OVA-conjugated KFE5 fibrils monitored by ThT intensity in the presence of (B) only Trypsin and (C) Trypsin-Chymotrypsin cocktail. *p < 0.05, **p < 0.01, by two-way ANOVA.
